# Leveraging antigenic seniority for maternal vaccination to prevent mother-to-child transmission of HIV-1

**DOI:** 10.1038/s41541-022-00505-w

**Published:** 2022-07-30

**Authors:** Ashley N. Nelson, Maria Dennis, Jesse F. Mangold, Katherine Li, Pooja T. Saha, Kenneth Cronin, Kaitlyn A. Cross, Amit Kumar, Riley J. Mangan, George M. Shaw, Katharine J. Bar, Barton Haynes, Anthony M. Moody, S. Munir Alam, Justin Pollara, Michael G. Hudgens, Koen K. A. Van Rompay, Kristina De Paris, Sallie R. Permar

**Affiliations:** 1 Human Vaccine Institute, Duke University Medical Center, Durham, NC USA; 2 Gillings School of Public Health and Center for AIDS Research, University of North Carolina at Chapel Hill, Chapel Hill, NC USA; 3 Molecular Genetics and Microbiology, Duke University School of Medicine, Durham, NC USA; 4 Department of Medicine, University of Pennsylvania, Philadelphia, PA USA; 5 California National Primate Research Center, University of California, Davis, CA USA; 6 Department of Microbiology and Immunology and Center for AIDS Research, School of Medicine, University of North Carolina at Chapel Hill, Chapel Hill, NC USA

**Keywords:** Protein vaccines, Antibodies, HIV infections

## Abstract

The development of a maternal HIV vaccine to synergize with current antiretroviral drug prophylaxis can overcome implementation challenges and further reduce mother-to-child transmission (MTCT) of HIV. Both the epitope-specificity and autologous neutralization capacity of maternal HIV envelope (Env)-specific antibodies have been implicated in decreased risk of MTCT of HIV. Our goal was to determine if heterologous HIV Env immunization of SHIV.C.CH505-infected, ART-suppressed female rhesus macaques (RMs) could boost autologous Env-specific antibodies. SHIV.C.CH505-infected female RMs (*n* = 12), began a daily ART regimen at 12 weeks post-infection (wpi), which was continued for 12 weeks. Starting 2 weeks after ART initiation, RMs received 3 monthly immunizations with HIV b.63521/1086.C gp120 or placebo (*n* = 6/group) vaccine with adjuvant STR8S-C. Compared to the placebo-immunized animals, Env-vaccinated, SHIV-infected RMs exhibited enhanced IgG binding, avidity, and ADCC responses against the vaccine immunogens and the autologous SHIV.C.CH505 Env. Notably, the Env-specific memory B cells elicited by heterologous vaccination were dominated by cells that recognized the SHIV.C.CH505 Env, the antigen of primary exposure. Thus, vaccination of SHIV-infected, ART-suppressed RMs with heterologous HIV Envs can augment multiple components of the antibody response against the Env antigen of primary exposure, suggesting antigenic seniority. Our results suggest that a universal maternal HIV vaccination regimen can be developed to leverage antigenic seniority in targeting the maternal autologous virus pool.

## Introduction

Significant strides have been made globally in the prevention of mother-to-child transmission (MTCT) of HIV. Over the past decade, expanded access to and uptake of ART during pregnancy has helped to reduce the risk of perinatal and postnatal MTCT of HIV to as low as 2%^1^. Despite this significant progress, ~150,000 infants became newly infected with HIV in 2019^1^ due to a number of limitations of an ART-based strategy, including acute maternal infection, late presentation to antenatal care, lack of maternal access, poor adherence to ART regimens (with increased risk for the emergence of drug-resistant mutants), and postpartum loss to follow-up^2–4^. Therefore, effective maternal immunization strategies in conjunction with the current ART-based prophylaxis can help to achieve a HIV-free generation.

In the absence of ART, less than half of infants will become infected, suggesting that there are maternal immune factors that provide partial protection against vertical transmission. Identifying these maternal immune correlates of decreased MTCT risk are critical to guiding the development of immune based strategies for prevention of MTCT of HIV. We previously reported that the magnitude of maternal IgG antibodies specific for HIV envelope (Env) variable loop 3 (V3), neutralization of tier 1 viruses, and the magnitude of CD4-binding site (CD4bs) blocking antibodies were associated with reduced risk of perinatal transmission among clade B, HIV-infected, ART naïve mothers (Women and Infant Transmission Study, WITS Cohort)^5^. Yet, among clade C virus-infected women from the Breastfeeding and Nutrition (BAN) cohort (*n* = 88) who received peripartum ART prophylaxis, maternal anti-variable loop 1 and 2-specific IgG and CD4bs-specific antibody responses trended towards being associated with increased risk of MTCT of HIV^6^. Furthermore, V3-specific antibodies isolated from a non-transmitting mother could neutralize co-circulating virus variants^5^, suggesting that antibodies with autologous virus neutralization capacity are protective in the setting of MTCT. Thus, temporary enhancement of maternal autologous virus neutralizing responses during pregnancy via immunization may be a strategy to further decrease HIV MTCT risk. Ideally, this would be achieved via implementation of a universal therapeutic vaccine.

As the autologous circulating virus pool varies widely among individuals and across geographical regions, a successful universal therapeutic maternal HIV vaccine would need to harness the concept of antigenic imprinting, boosting maternal autologous virus-specific antibody responses in the setting of closely related immunogens. As original antigenic sin generally implies the negative consequences of pre-existing immunity, a more recent model of immune imprinting has been adopted, termed “antigenic seniority”^7,8^. Under this model, the first exposure to an antigen elicits antibodies that take a “senior position” in the immune framework, and future infections/immunizations boost these pre-existing antibody responses while also producing their own lower magnitude antibody responses^7,8^. Exploiting this model of antigenic seniority will be key in the development a universal vaccine for the prevention of MTCT of HIV.

Non-human primates have served as an invaluable model for advancing our understanding of MTCT of HIV, and evaluation of strategies for prevention^9,10^. In this study we evaluated the ability of a HIV Env vaccine regimen to boost potentially protective maternal Env-specific antibody responses in SHIV-infected, ART-suppressed, non-pregnant female rhesus macaques (RMs). We sought to (1) determine whether a heterologous HIV Env vaccine regimen could boost IgG responses specific for the autologous (challenge) virus Env antigen; and (2) assess if HIV Env vaccination could enhance autologous virus neutralization and maternal humoral immune responses previously identified in human cohorts as associated with reduced MTCT risk. A maternal vaccine that achieves these goals could synergize with current ART regimens as an added layer of protection to further reduce MTCT, to levels not achieved by ART alone.

## Results

### Overall experimental design

The current study was designed to test the ability of a heterologous HIV Env vaccine regimen to boost autologous virus-specific antibody responses that may be useful in reducing the risk of vertical virus transmission. Due to current World Health Organization (WHO) recommendations, most HIV-infected pregnant women will be on an ART regimen throughout pregnancy, thus, our immunizations were administered in the setting of a daily ART. Furthermore, this vaccine regimen was designed as a potential therapeutic for HIV-infected pregnant women to simultaneously address challenges in ART treatment failures while boosting maternal antibody responses previously associated with decreased MTCT risk. As such, we designed a nonhuman primate study to model the setting of maternal HIV infection and initiation of ART during the gestational period, combined with Env vaccination to elicit potentially protective immune responses against the autologous, circulating virus. Due to the limited availability and expense of pregnant macaques, this study was performed in non-pregnant female macaques to demonstrate proof-of-concept.

#### Virological outcome of ART and vaccine interventions

**Fig. 1:**
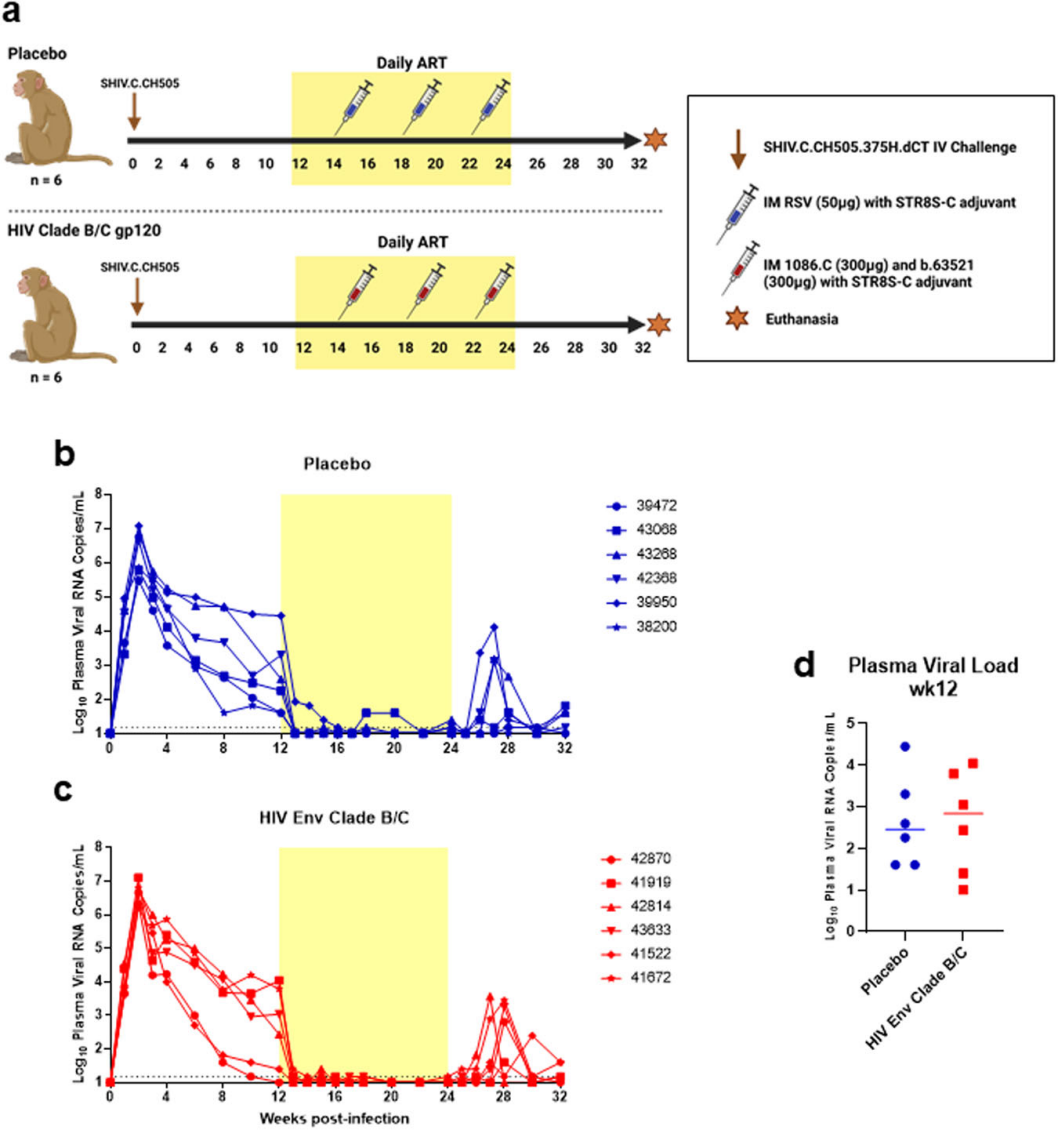
Infection, treatment, and immunization schedule for study of vaccine-elicited responses in SHIV-infected, ART-treated female monkeys to reduce MTCT. **a** Study design schematic. Twelve female RMs were infected with SHIV.C.CH505 (brown arrow), started on a daily ART regimen at 12 wpi (yellow box). Six RMs received either a placebo vaccine (blue syringes) or a HIV Env combined clade B/C gp120 vaccine (red syringes). ART was discontinued at 24 wpi, and RMs were monitored for an additional 8 weeks. Plasma viral RNA kinetics through 32 wpi for placebo vaccinated (**b**) and HIV Env Clade B/C gp120 (**c**) vaccinated RMs. Plasma vRNA load at 12 wpi, prior to ART start, in placebo (blue) and HIV Env (red) vaccinated RMs (**d**). Medians are represented by the horizontal lines.

Twelve female, adult rhesus macaques (RMs) were infected intravenously with SHIV.C.CH505, and started on a daily ART regimen at 12 weeks post-infection (Fig. 1a). Prior to vaccination, two groups of six were balanced based on plasma VL at 12 wpi (Table 1; Fig. 1b–d). Six RMs were assigned to the placebo vaccine group, and the remaining six received an HIV Env combined Clade B/C vaccine (Fig. 1a). Plasma viral load kinetics were monitored over the course of the study and are shown in Fig. 1b (Placebo) and 1c C (HIV Env Clade B/C). Four animals (*n* = 2/group) did exhibit spontaneous control of viral replication (<150 copies vRNA/mL) prior to ART start (Table 1; Fig. 1b–d), which is not uncommon in NHP models of SHIV infection^11–13^. One spontaneous controller was positive for the Mamu-A*01 MHC I allele associated with attenuated disease progression and increased control of SIV/SHIV replication^14,15^, while one spontaneous controller had a higher frequency of CD8 + T cells at baseline compared to the rest of the cohort (Table 1). Viral loads declined to below the limit of detection within 2–3 weeks of ART initiation, with viral blips observed in one RM in the placebo group from weeks 18–20. Upon cessation of therapy, viral rebound, defined as HIV RNA levels of >150 copies/mL (10X detection limit of the assay), occurred in 3 of 6 RMs in the placebo group and 5 of 6 HIV Env vaccinated RMs (Table 2). Among the RMs that exhibited viral rebound, time to rebound was similar between the two groups (*p* = 0.60). The median time to viral rebound was 3 weeks (range: 2–3 weeks) in the placebo group and 4 weeks (range: 3–4 weeks) in the vaccinated group (Table 2). Lastly, of the four spontaneous controllers, only two exhibited viral rebound after ART cessation.

**Table 1:** MHC status, immune parameters and viral load at baseline and prior to ART initiation in vaccine and placebo recipient SHIV-infected, ART-treated RMs.

	Animal ID	A*01 Status^a^	CD4 Frequency (%) at Baseline	CD8 Frequency (%) at Baseline	Plasma VL^b^at Baseline	CD4 Frequency (%) at ART Initiation	CD8 Frequency (%) at ART Initiation	Plasma VL^b^at ART Initiation
**Placebo**	39472	–	36.7	25.7	<15	33.4	28.3	40
	43068	–	34.3	12.3	<15	35.3	18.5	180
	43268	–	38.3	17.2	<15	33.8	14.6	390
	42368	–	28.9	15.4	<15	28.5	20.5	2,000
	39950	–	32.5	23.4	<15	29.8	29.2	28,000
	38200	–	29.6	18.1	<15	28.3	22.3	40
**HIV Env Clade B/C**	42870	+	38.2	13.7	<15	44.9	15.9	<15
	41919	–	37.7	9.8	<15	35.3	12.6	11,000
	42814	–	39.9	16.0	<15	42.0	17.7	270
	43633	–	28.5	11.6	<15	25.3	16.2	1,100
	41522	–	44.2	39.9	<15	38.3	43.9	25
	41672	n/a	34.7	25.7	<15	29.9	24.3	6,200

^a^Mamu*A01 status. (+) = positive, (−) = negative, n/a = not applicable as data is not available.

^b^Plasma VL indicated as vRNA copies/ mL.

**Table 2:** Time to viral rebound after discontinuation of ART and peak viral load at rebound.

	PTID	Time to viral reboud (weeks)	Peak VL at rebound
**Placebo**	39472	No rebound	<150
	43068	No rebound	<150
	43268	3	1500
	42368	3	1300
	39950	2	13,000
	38200	No rebound	<150
**HIV Clade B/C**	42870	4	650
	41919	No rebound	<150
	42814	3	3700
	43633	4	1800
	41522	4	250
	41672	3	2800

### HIV Env vaccination enhanced plasma gp120-specific IgG binding responses with increased avidity

**Fig. 2:**
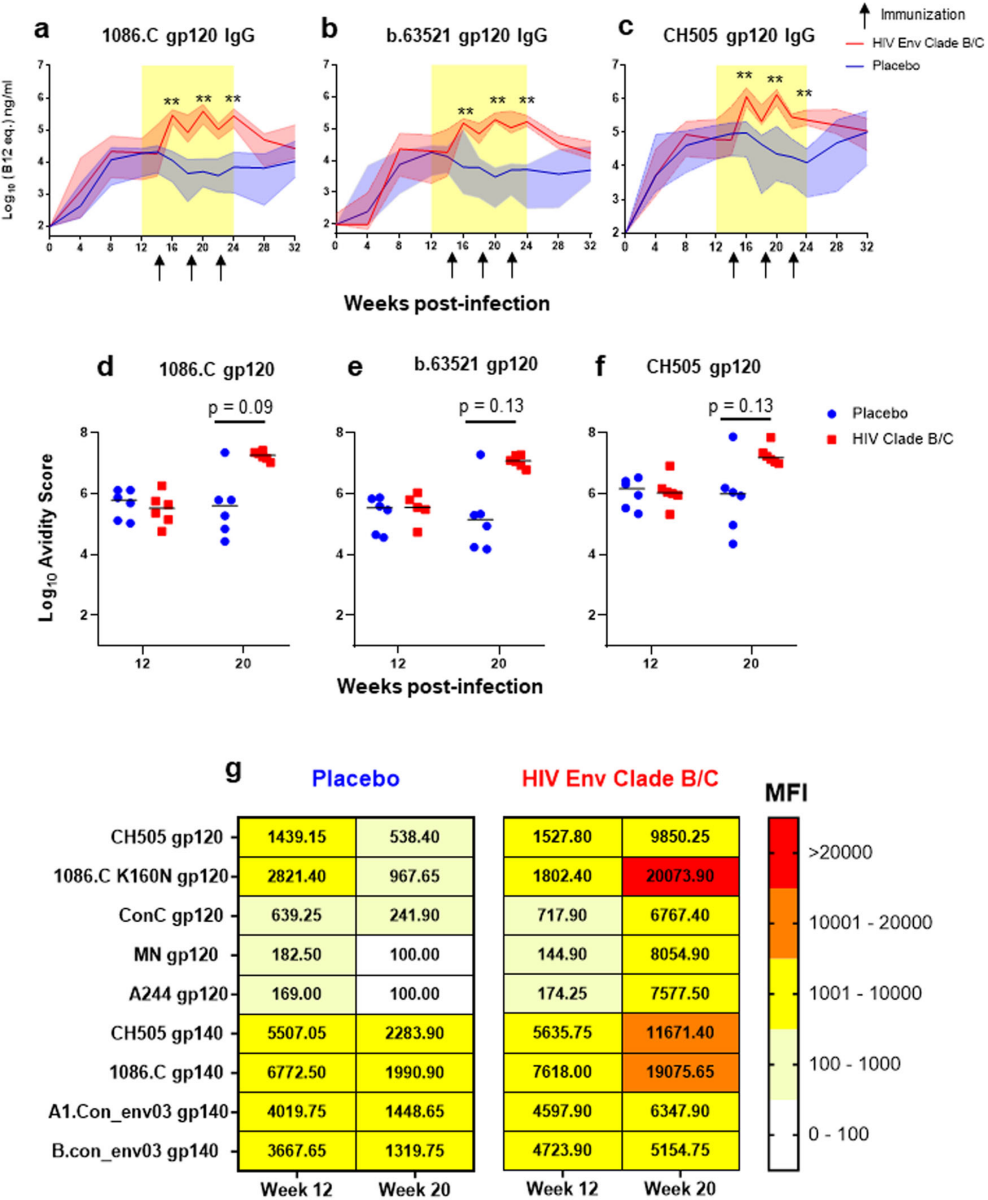
Plasma gp120-specifc IgG antibody binding kinetics, avidity, and breadth. Plasma gp120 IgG binding responses to vaccine antigens 1086.C (**a**) and b.63521 (**b**), and challenge virus antigen, CH505 (**c**) in placebo (blue) and Env vaccinated (red) macaques. Bold lines represent the median, and the range is depicted by the shaded area; black arrows indicate time points at which immunizations were administered (14, 18, and 22 wpi). Antibody avidity was assessed against vaccine and challenge antigens at week 12, pre-ART/immunization, and at week 20 when IgG gp120 binding peaked in the Env vaccinated group (**d**–**f**). Dot plots represent avidity score, each dot represents one animal, and medians are represented by horizontal lines (**d**–**f**). Statistical analysis was performed using Wilcoxon rank-sum tests with exact *p*-values to compare gp120-specifc IgG binding (Supplementary Table 3) and avidity (Supplementary Table 4) responses between vaccinated and placebo RMs, followed by FDR adjustments for multiple comparisons. **unadjusted *p* < 0.01. FDR adjusted *p*-values are reported in the dot plots. See Supplementary Table 3 and 4 for both unadjusted *p* and FDR_p for all comparisons. **g** Comparison of gp120 and gp140 IgG binding breadth at pre-ART/immunization, week 12, and at peak IgG binding post-immunization, week 20. Data shown are median MFI for each antigen for placebo (left panel) and HIV Env vaccinated (right panel) RMs.

Plasma IgG binding responses against vaccine antigens, 1086.C and b.63521 gp120 (Fig. 2a, b), and the challenge virus antigen, CH505 gp120 (Fig. 2c), were measured over the course of the study. Prior to treatment and immunization, the magnitude and kinetics of binding responses were similar between both groups. During the treatment period, the placebo vaccinated group exhibited a continuous decline in gp120-specific binding responses (Fig. 2a–c), consistent with previous reports of ART-associated decline in HIV/SHIV-specific antibody responses^16,17^. Two weeks following each immunization (i.e. at weeks 16, 20, and 24), the magnitude of IgG binding was consistently higher in vaccinated RMs compared to placebos against 1086.C (Fig. 2a), b.63521 (Fig. 2b), and CH505 gp120s (Fig. 2c). After ART was suspended, the gp120-binding IgG responses began to return to levels similar to what was observed prior to treatment and immunization, confirming observations from previous studies reporting an increase in HIV-specific antibodies following ART cessation in humans and non-human primate models^11,18,19^. In all, HIV Env vaccination resulted in temporary enhancement of vaccine and challenge virus gp120-specific IgG binding responses in SHIV-infected, ART-suppressed RMs (Fig. 2a–c).

The avidity of HIV Env-specific IgG antibodies to vaccine and challenge virus gp120 were measured by surface plasmon resonance (SPR) prior to treatment/immunization (week 12) and 2 weeks after the 2nd immunization (week 20), when peak binding responses occurred in the Env vaccinated cohort (Fig. 2d–f). Avidity scores were generally higher in the Env vaccinated RMs at week 20 against vaccine (1086.C gp120: FDR *p* = 0.09; b.63521: FDR *p* = 0.13; Fig. 2d, e) and challenge virus (FDR *p* = 0.13; Fig. 2f) gp120 antigens compared to the placebo group. Interestingly, one RM in the placebo vaccinated group exhibited avidity scores comparable to that observed in the vaccinated group across all three antigens.

In addition, we used a binding antibody multiplex array (BAMA) to assess cross-clade gp120 and gp140 breadth at weeks 12 and 20 post-infection (Fig. 2g). Both groups developed breadth of plasma Env-specific IgG responses by 12 wpi. However, the median Env-specific IgG binding responses increased across all antigens at 20 wpi in the Env vaccinated RMs, while the majority of those responses declined in the placebo group (Fig. 2g).

### HIV Env-vaccinated RMs exhibit plasma epitope specific responses of greater magnitude and breadth

**Fig. 3:**
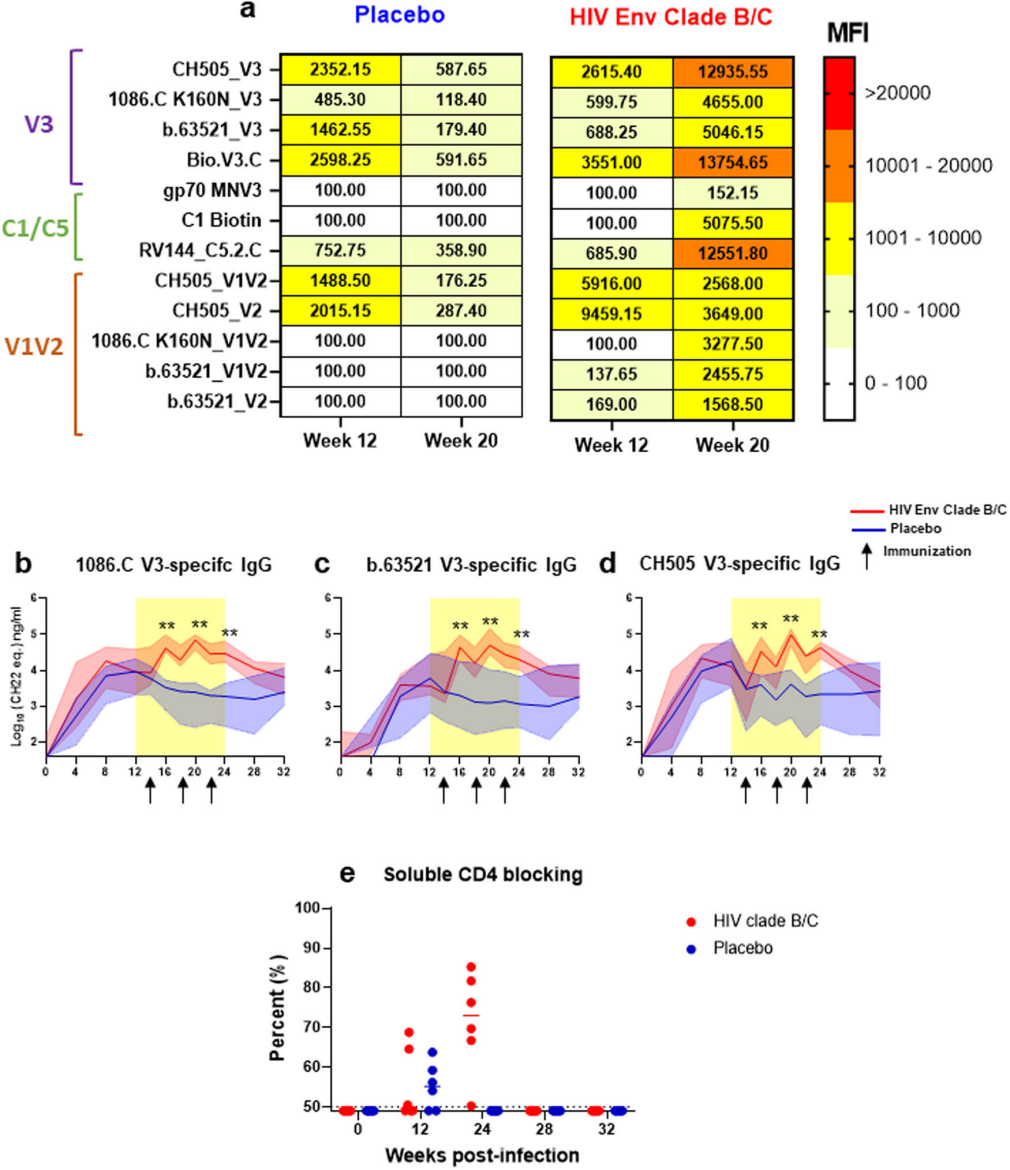
Epitope specificity of Env-specific IgG responses. Plasma IgG specificity to a panel of HIV Env linear and conformational epitopes at pre-ART/immunization, week 12, and at peak IgG binding, week 20 post-infection (**a**). Data shown are median MFI for each antigen. Level of V3-specific IgG binding against vaccine (**b**, **c**), and challenge virus antigen (**d**) measured by quantitative ELISA. Bold lines represent the median, and the range is depicted by the shaded area. Black arrows indicate time points at which immunizations were administered (14, 18, and 22 wpi). Statistical analyses were performed using Wilcoxon rank-sum tests with exact *p*-values to compare V3-specifc IgG responses between vaccinated and placebo RMs, followed by FDR adjustments for multiple comparisons. **unadjusted *p* < 0.01. **e** Plasma blocking of soluble CD4-1086.C gp120 interactions. Each dot represents one animal, and medians are represented by horizontal lines.

Some studies have suggested that maternal Abs against vulnerable regions of the HIV envelope (Env), such as the V1V2-apex, V3, and CD4-binding site (CD4bs) blocking antibodies are associated with decreased MTCT risk of HIV^20,21^. To determine the impact of HIV Env vaccination on the breadth and epitope specificity of the plasma Env-specific IgG responses, we assessed binding to various HIV Env linear and conformational epitopes. Antibody epitope specificity in the two groups were measured at 12 wpi, a pre-ART/immunization time point, and at 20 wpi, the time point at which gp120-specific IgG binding responses were highest in the vaccinated cohort. For both groups, anti-V3 and CH505 V1V2 binding responses were dominant at week 12 (Fig. 3a). Yet, by week 20 (2 weeks post- 2nd immunization), the HIV Env vaccinated cohort exhibited an increase in plasma IgG binding responses to all epitopes, across multiple virus clades including those representing the vaccine and challenge virus antigens (Fig. 3a). At week 20 post-infection, the dominant responses in the vaccinated cohort were against the linear V3 and C5 regions. As a previous maternal humoral immune correlate study of subtype B HIV-infected, ART-naïve mothers reported that the magnitude of maternal V3-specific IgG antibodies were associated with reduced risk of MTCT^5^, we further assessed the kinetics of plasma vaccine and challenge virus strain V3-specific binding responses (Fig. 3b–d). While V3-specific IgG responses were similar in both groups prior to immunization, these responses increased in the vaccinated cohort over the course of treatment/immunization compared to the placebo group (Fig. 3b–d). Lastly, we measured plasma CD4-binding site (CD4bs) blocking antibody responses and observed greater blocking of soluble CD4 among Env immunized RMs by week 24 (2 weeks post 3rd immunization) (Fig. 3e). Thus, Env vaccination in the setting of ART could boost IgG responses previously, yet not consistently, associated with decreased MTCT risk^5,6^.

### Autologous Env-specific ADCC antibody titers are enhanced by HIV Env vaccination of SHIV-infected, treated animals, yet autologous virus neutralization is not

**Fig. 4:**
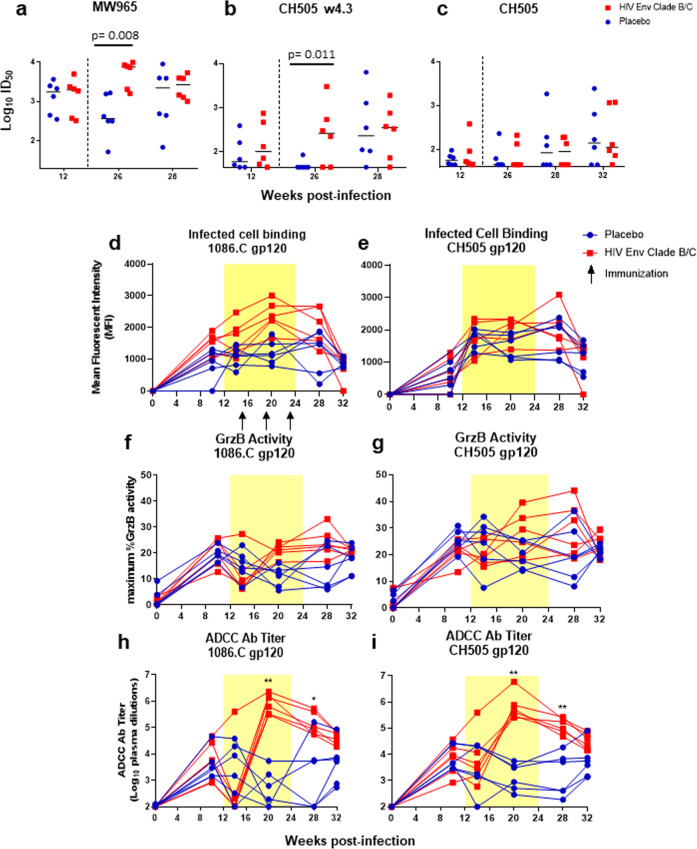
Functional antibody responses elicited in vaccinated and placebo recipient SHIV-infected, ART-treated rhesus monkeys. Plasma neutralizing activity against tier 1 viruses, MW965 (**a**) and CH505 w4.3 (**b**) and the autologous CH505 (**c**) virus. Medians are indicated as horizontal lines, vertical dotted line indicates ART stop. Levels of infected cell binding expressed as Mean Fluorescent Intensity to a panel of HIV-1 1086.C or CH505.TF IMC-infected cells over time for each animal are shown (**d**, **e**). Maximum granzyme B activity (**f**, **g**) and the plasma dilution endpoint ADCC antibody titers (**h**, **i**) against 1086.C and CH505 gp120-coated target cells over time for each animal are shown. Statistical analyses were performed using Wilcoxon rank-sum tests with exact *p*-values to compare plasma IgG neutralization (**a**–**c**) and ADCC antibody titers (**h**, **i**) between vaccinated and placebo RMs, followed by FDR adjustments for multiple comparisons. *unadjusted *p* < 0.05, **unadjusted *p* < 0.01. FDR adjusted *p*-values are reported in the dot plots. See Supplementary Table 3 for both unadjusted *p* and FDR_p for all comparisons.

The development of plasma antibody-mediated antiviral effector functions was compared between vaccinated and placebo RMs, including neutralization, antibody-dependent cell-mediated cytotoxicity (ADCC), and infected cell binding. Plasma HIV-1 neutralization was assayed in TZM-bl cells against the clade C tier 1 viruses MW965 and CH505 w4.3, and the autologous, CH505 transmitted/founder virus at week 12 (pre ART/immunization) and at weeks 26, 28, and 32. Tier 1 virus neutralization responses were similar between the two groups at 12 wpi (FDR *p* = 1 and 0.59 for MW965 and CH505 w4.3, respectively; Fig. 4a, b), however, these responses were more rapidly induced and of greater potency in the vaccinated RMs at 26 wpi (2 weeks post-ART cessation) (MW965 ID_50_ range vaccinated: 1595–9822, placebo: 69–1644, FDR *p* = 0.008; CH505 w4.3 ID_50_ range vaccinated: 45–3028, placebo:45—86, FDR *p* = 0.01). Interestingly, by 28 wpi virus neutralization responses in the vaccinees had waned and were similar between the two groups (FDR *p* = 0.29 and 1, MW965 and CH505 w4.3, respectively; Fig. 4a, b). In contrast, neutralization responses against the CH505 challenge virus strain were similar between the two groups pre-ART/immunization and post-ART, although there was a slight increase in potency of this response at weeks 28 and 32 post-infection (6 and 10 weeks post-3rd immunization) (Fig. 4c). Thus, heterologous HIV Env vaccination elicited an increase in tier 1 virus neutralization, but not autologous virus neutralization following the treatment and immunization period.

The ability of plasma IgG to bind HIV-infected cells expressing 1086.C or CH505 Env antigens was measured by the MFI of bound antibodies, (Fig. 4d–e). Overall, the ability of antibodies to bind infected cells were similar between the two groups. Similarly, the maximum observed ADCC activity was comparable between the two groups (Fig. 4f, g). However, ADCC antibody titers specific for 1086.C gp120 were boosted in vaccinated animals at week 20 (2 weeks post 2nd immunization) compared to the placebo recipient animals (median ADCC titers were 9.8 × 10^5^ and 4.0 × 10^2^, FDR *p* = 0.005 vaccinated and placebo, respectively; Fig. 4h). Similarly, CH505 gp120-specific ADCC titers were boosted in vaccinated animals at week 20 (median ADCC titers were 4.7 × 10^5^ and 2.0 × 10^3^, FDR *p* = 0.004, vaccinated and placebo, respectively; Fig. 4i). ADCC titers remained higher in the vaccinated RMs following the treatment and immunization period at 28 wpi (FDR *p* = 0.046 for 1086.C; and FDR *p* = 0.004 for CH505). Thus, HIV Env immunization in SHIV-infected, ART suppressed RMs considerably boosted the quantity but not the overall activity of antibodies capable of mediating ADCC.

### Heterologous HIV Env vaccination in the setting of ART boosts pre-existing autologous virus-specific antibody responses

**Fig. 5:**
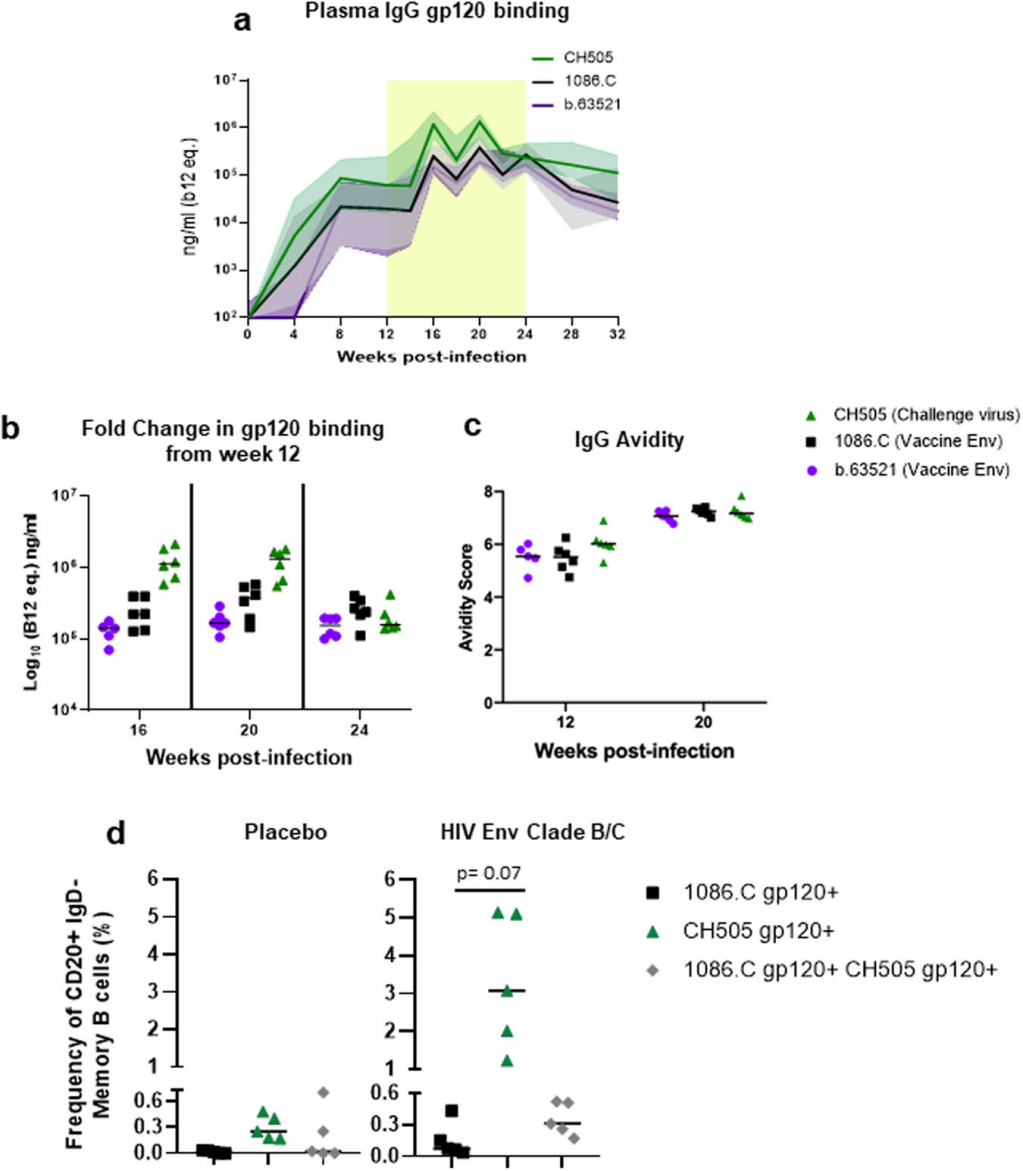
Heterologous Env vaccination boosts pre-existing autologous Env-specific B cell responses compared to vaccine-specific responses among vaccinated RMs- evidence for antigenic seniority. IgG binding responses specific for vaccine and challenge virus antigens were compared among Env vaccinated RMs (**a**). To evaluate changes in IgG binding responses among the vaccinated cohort across all three antigens, the fold change (**b**) from week 12 (pre-ART/immunization) were compared for each antigen at 16, 20, and 24 wpi (2 weeks post each immunization). Antibody avidity, measured by SPR, across all three antigens were also compared among vaccinees (**c**). Frequency of CH505 and 1086.C gp120-specific memory B cells at week 24 post-infection in HIV Env vaccinated and placebo recipient RMs (**d**). Each data point represents one animal, and medians are indicated as horizontal lines. Statistical analyses were performed using Wilcoxon signed-rank tests with exact *p*-values to compare Env vaccinated RMs’ plasma CH505 gp120 IgG binding and avidity responses, and Env vaccinated RMs’ CH505 gp120-specific memory B cells to 1086.C and b.63521-specific responses, followed by FDR adjustments for multiple comparisons. See Supplementary Table 5 for unadjusted p and FDR_p for all comparisons.

To determine if the antibody responses elicited among the Env vaccinated RMs exhibited evidence of antigenic seniority, we compared the vaccine and challenge virus Env-directed antibody binding and functional responses within this group. Env vaccination boosted antibody binding responses specific for all three antigens. Binding responses to the autologous CH505 gp120 antigen were slightly higher compared to the vaccine-specific Env binding responses at week 16, 2 weeks post the 1st immunization (CH505 versus 1086.C: FDR *p* = 0.06; for CH505 versus b.63521: FDR *p* = 0.06; Fig. 5a). Similarly, CH505 gp120 binding responses remained higher at week 20 (CH505 versus 1086.C: FDR *p* = 0.06; for CH505 versus b.63521: FDR *p* = 0.06; Fig. 5a). By week 24, 2 weeks after the 3rd immunization, CH505 gp120 binding responses were only higher than those against b.63521 gp120 (FDR *p* = 0.06; Fig. 5a). Furthermore, median fold changes in binding responses from week 12 were slightly higher for CH505 and 1086.C gp120 at weeks 16 and 20, which are both clade C antigens, compared to b.63521 gp120. (Fig. 5b). There was an increase in antibody avidity responses specific to the vaccine and challenge virus antigens from week 12 to 20, however, these responses were similar in magnitude across all three antigens (20 wpi: CH505 versus 1086.C, FDR *p* = 0.31; CH505 versus b.63521, FDR *p* = 0.17; Fig. 5c). Finally, we determined whether heterologous Env vaccination elicited memory B cell responses generated from the primary, SHIV.C.CH505, exposure. The frequency of CH505 Env-specific memory B cell responses measured at 24 wpi (2 weeks post 3rd immunization; Supplementary Fig. 1) was higher compared to 1086.C gp120-specific responses among the vaccinated RMs (FDR *p* = 0.07; Fig. 5d). Of note, CH505 Env-specific memory B cell responses at 24 wpi were higher in the vaccinated RMs compared with placebo. These results demonstrate that heterologous Env vaccination in the setting of ART can temporarily enhance autologous virus-directed antibody responses. Furthermore, these data support a role for exploiting the antigenic seniority model for development of a maternal HIV vaccine regimen, in which a universal heterologous HIV Env vaccine administered in the setting of infection and ART can boost maternal autologous virus-directed antibody responses.

### Evolution of HIV Env sequences upon rebound in vaccinated and unvaccinated SHIV-infected rhesus monkeys

As viral escape from maternal neutralizing antibodies is associated with vertical virus transmission^22–25^, we investigated whether maternal vaccination led to distinct evolution of the viral reservoir compared to the placebo group. Neighbor-joining phylogenetic trees were generated to visualize evolutionary relationships and sequence diversity of the *env* gene of viruses from the pre-ART and rebound time points in four (*n* = 2/group) Env-vaccinated and control-vaccinated RMs that demonstrated viral rebound, summarized in Supplementary Table 1. Both pre-ART/immunization and rebound *env* single genome amplicons (SGA) were successfully obtained from 2 RMs in each group. Unlike in the pre-ART samples, recovery of full-length *env* genes from plasma at rebound was limited, seemingly due to frequent large deletions within the *env* gene suggesting that the rebound virus pool includes defective viruses^26–28^. In all animals, pre-ART/immunization and rebound viral *env* sequences clustered separately, suggesting divergent evolution of viruses within each animal (Supplementary Fig. 2). Highlighter plot analyses were performed comparing pre-ART/immunization and rebound *env* sequences to the SHIV.C.CH505 challenge virus sequence in order to visualize specific mutations in the *env* gene. This analysis revealed that while *env* gene evolution within each animal generally followed similar patterns of mutation, there is some sequence diversity between animals (Supplementary Figs. 3 and 4). A highlighter plot comparing rebound *env* sequences across the vaccine groups further demonstrates that there is not a distinct pattern of rebounding virus evolution in Env-vaccinated compared to control-vaccinated monkeys (Supplementary Fig. 4). Overall, there was no evidence of divergent evolution of the rebound virus in Env-vaccinated animals compared to the placebo recipients. Thus, there appeared to be minimal impact of Env vaccination on the evolution of rebounding virus.

### Correlates of viral rebound among HIV Env vaccinated and unvaccinated SHIV-infected RMs

Although heterologous HIV Env vaccination boosted antibody responses against the autologous Env, these responses did not impact time to viral rebound compared to placebo vaccinated RMs. This observation was not surprising as ART was initiated at 12 wpi, and the viral reservoir is already established by this time^29,30^. To define the virologic parameters and/or antibody responses associated with time to viral rebound among all RMs, a Spearman’s rank-order correlation was estimated between time to viral rebound and viral load at week 12, CH505-specific ADCC antibody titers (weeks 10, 20, and 28), CH505 autologous virus neutralization (weeks 12, 26, and 28), and CH505-specific avidity score (weeks 12 and 20). Of these parameters, autologous virus neutralization at week 28 was found to have the strongest inverse correlation with time to viral rebound (Supplementary Fig. 5).

## Discussion

Additional intervention strategies to complement current ART regimens are critical in order to achieve the WHO and UNAIDS Global Plan goal of eliminating MTCT of HIV^31^. Here we designed a nonhuman primate study to model HIV Env vaccination in the setting of maternal HIV infection and recent initiation of ART during the gestational period, and assessed the ability of vaccination to elicit potentially protective immune responses against the autologous, circulating virus. Furthermore, this study functions as a proof-of-principle model for assessing the ability of this heterologous Env vaccination strategy to elicit immune responses identified in human cohorts to be associated with decreased risk of MTCT of HIV. Overall, we have demonstrated that Env vaccination in SHIV-infected, ART-suppressed RMs enhanced IgG responses against b.63521 and 1086.c vaccine immunogens, as well as against the challenge virus Env, SHIV.C.CH505, when compared to the placebo group. Despite the enhancement of Env-specific antibody responses, HIV Env vaccination did not prevent or significantly delay time to viral rebound which is likely attributed to late ART initiation (at 12 wpi). HIV establishes latent reservoirs extremely early after infection, with vertical transmission studies suggesting this could occur as early as 30 h post-infection^29,32^, and reactivation of viral reservoirs following ART cessation is believed to be responsible for rebound viremia^33,34^. To this end, it is not surprising that our data corroborates that of previous reports that therapeutic vaccination of ART-suppressed, SIV-infected RMs or HIV-infected persons does not significantly impact the time to viral rebound between vaccinated and control groups, although humoral immune responses are boosted^35,36^.

In the absence of any maternal ART intervention, less than half of infants become HIV infected^37^, signifying the potential for maternal immune factors to provide partial protection against the vertical or post-natal transmission of HIV. Maternal anti-gp120 antibodies have been inversely associated with MTCT vertical transmission risk^38^. Moreover, several lines of evidence suggest that maternal Abs targeting specific sites of the HIV envelope (Env) are associated with protection against MTCT of HIV^20,21^. The magnitude of maternal IgG antibodies specific for Env third variable loop (V3)^5^ and increased levels of CD4-binding site (CD4bs) blocking antibodies are reported to be associated with reduced risk of MTCT in a cohort of subtype B, HIV-infected, ART-naïve mothers^5,39^. We have shown that immunization in the setting of SHIV infection and ART suppression could enhance both V3-specific and CD4bs blocking antibody responses in Env immunized macaques compared to the placebo group. Notably, we demonstrated that V3-specific responses targeting the antigen of primary exposure, SHIV.C.CH505, can be temporarily boosted in response to a heterologous immunization regimen. The magnitude of maternal V1V2-apex targeting antibodies was associated with decreased MTCT risk in clade C-infected mothers, receiving antiretroviral prophylaxis^6^. To this end, we demonstrated that autologous V1V2-specific responses were also boosted in HIV Env immunized RMs. While maternal Env-specific Abs targeting the membrane-proximal external region (MPER) of gp41 have also been shown to be associated with reduced risk of vertical HIV transmission among HIV subtype C-infected mothers^40^, this response was not explored in our study.

In the setting of MTCT of HIV, most infant infections are established by a single transmitted-founder (T/F) variant indicating a selective virus genetic bottleneck is involved in transmission^41,42^. While the specific role of maternal autologous virus neutralizing IgG antibodies remains unclear, some studies suggest that maternal antibodies select for neutralization escape variants that initiate infection in the infant^42,43^. In the setting of peripartum HIV transmission, infant T/F viruses were more neutralization resistant against paired maternal plasma than non-transmitted maternal virus variants^22^. Furthermore, linear V3-specific antibodies isolated from a non-transmitting, HIV-infected mother from a WITS cohort study neutralized her own circulating virus variants^5^, suggesting a role for maternal autologous virus neutralizing antibodies in protection from MTCT. Thus, an effective therapeutic maternal HIV vaccine for MTCT prevention would ideally be both universal and able to boost autologous virus-directed antibody responses. As such, we explored whether our heterologous HIV Env immunization regimen would elicit antibody responses that follow a pattern of antigenic seniority, a model adopted in many influenza immune response studies as a refinement to the original antigenic sin (OAS) hypothesis^7,44–46^. This more recent model of OAS dictates that strains of primary and/or early exposure are given a more ‘senior’ antigenic position in our immune repertoire, and that each subsequent strain takes a more junior position in the response. Thus, a key distinction of this new model is that every new strain gets a place in the immune hierarchy, not only the strain of first exposure^7^. Our heterologous Env immunization regimen did not enhance autologous virus neutralization responses which is likely attributed to control of viral replication in many of the RMs by 12 wpi (pre-ART/immunization). Additionally, as autologous virus neutralization was measured against the transmitted/founder virus inoculum stock, our observations may not be representative of autologous virus neutralization responses developed against circulating virus pools. While antibody binding responses targeting the autologous antigen were minimally boosted in comparison to vaccine-specific responses, autologous, SHIV.C.CH505 gp120-specific memory B cells were elicited at higher frequencies after 3 immunizations compared to the 1086.C gp120-specific memory B cells, suggesting a role for antigenic seniority in our model.

As our heterologous Env vaccine regimen failed to elicit potent autologous virus neutralization responses, alternative approaches will need to be considered for potent B cell stimulation to achieve enhancement of this response in ART-suppressed, HIV-infected pregnant women. Although, elicitation of high titer, potent HIV Env-specific antibody responses by vaccination comes with the potential risk of driving viral escape variants in maternal plasma that could contribute to (1) a higher frequency of viral rebound among vaccinees and (2) enhanced fitness for transmission to the infant^22–24^ Therefore, we evaluated the impact of our vaccine regimen on autologous virus evolution. Sequencing analysis of isolated *env* genes from pre-ART and rebound did not reveal evidence of selection pressure, or acquisition of specific mutations in vaccinated RMs compared to placebo. However, our analyses were limited by the inability to isolate enough Env sequences (>20) by single genome amplification from rebound time points to have >90% confidence that our sampling reflects an adequate representation of the diversity of the autologous virus pool^23,47^.

Assessment of non-neutralizing antibody functions in our study revealed that titers of ADCC-mediating antibodies against both autologous and vaccine Envs were boosted in vaccinated RMs when compared to the placebo group. Yet, it remains to be determined if enhancement of these responses would contribute to reduction in MTCT risk. In NHP models, passive immunization with hyperimmune serum that did not neutralize the challenge virus but had high ADCC activity protected newborn rhesus macaques from an oral challenge of pathogenic simian immunodeficiency virus (SIV), supporting a role for ADCC in reducing MTCT^48,49^. In human studies, breast milk Env-specific IgG responses with ADCC activity were associated with reduced MTCT risk among a cohort of HIV clade A virus-infected Kenyan women^50^, however neither breast milk nor plasma ADCC responses were associated with postnatal transmission risk among a cohort of clade C HIV-infected mothers^51^. Moreover, a recent study of the BG505-MG505, mother-infant transmitting pair found that while maternal autologous virus variants were mostly resistant to neutralization by maternal V3-specific antibodies present near the time of transmission, these antibodies exhibited potent autologous ADCC activity^52^. Despite these disparate findings, these studies further highlight a role for antibody specificity and function in the vertical transmission bottleneck. Furthermore, ADCC antibody activity in HIV-infected infants has been associated with reduced mortality risk^53^. Cumulatively, these findings reveal the need for further investigation into the role of ADCC-mediating antibodies in preventing MTCT of HIV.

Overall, our model has demonstrated that vaccination of SHIV-infected RMs with heterologous HIV Envs in the setting of ART can temporarily enhance IgG responses targeting the antigen of primary exposure, SHIV.C.CH505, as well as ADCC-mediating antibodies. As pregnancy can drastically alter the maternal immune environment, future studies in pregnancy models are needed to determine if vaccination can elicit similar protective responses in pregnant women. Nonetheless, our results suggest that a universal maternal HIV vaccine regimen can be developed to boost antibodies that target the maternal autologous virus pool, and elicit previously identified humoral immune correlates of reduced MTCT risk in humans. More importantly, HIV Env immunization was able to overcome the decline in Env-specific antibody responses associated with daily ART highlighting the potential for a therapeutic vaccine which can overcome challenges associated with implementation of daily ART regimens.

## Methods

### Animal care and sample collection

Type D retrovirus-, SIV- and STLV-1 free Indian rhesus macaques (RM) (*Macaca mulatta*) were maintained in the colony of California National Primate Research Center (CNPRC, Davis, CA). Adult female rhesus macaques were utilized in this study and ranged from 4 to 10 years of age as previously described^54^. Animals were maintained in accordance with the American Association for Accreditation of Laboratory Animal Care standards and The *Guide for the Care and Use of Laboratory Animals*^55^. For sample collections, animals were sedated with ketamine HCl (Parke-Davis) injected at 10 mg/kg body weight. EDTA-anticoagulated blood was collected via peripheral venipuncture. Plasma was separated from whole blood by centrifugation, and PBMCs were isolated by density gradient centrifugation using Ficoll^®^-Paque (Sigma) or Lymphocyte Separation Medium (MP Biomedicals). DNA extracted from splenocytes was used to screen for the presence of the major histocompatibility complex (MHC) class I allele Mamu-A*01 using a PCR-based technique (Table 1). All protocols were reviewed and approved by the University of California at Davis Institutional Animal Care and Use Committee (IACUC) prior to the initiation of the study.

### SHIV challenge, treatment, and immunization

To generate SHIV.C.CH505.375H.dCT, the SIVmac766 clone (GenBank accession no. KU955514) was modified by replacing the SIV genome between the stop codon of vpr and start codon nef with a linker containing the unique restriction enzyme sites AgeI and AclI. A complete HIV-1 CH505 tat/rev/vpu/env (gp160) cassette was inserted^56^. The SHIV.C.CH505.375H.dCT challenge stock (provided by Dr. George M. Shaw, University of Pennsylvania) was prepared by infecting primary activated Indian rhesus macaque CD4 + T cells and 7–14 days later culture supernatants were pooled. Virus titers were determined in TZM-bl cells, yielding 6.8 × 10^6^ TCID_50_ /ml.

Twelve, female adult rhesus monkeys were challenged intravenously with SHIV.C.CH505 at a dose of 3.4 × 10^5^ TCID_50_. At 12 weeks post-infection, RMs were started on a daily, subcutaneous ART regimen consisting of a co-formulation containing 5.1 mg/kg tenofovir disoproxil fumarate (TDF), 40 mg/kg emtricitabine (FTC) and 2.5 mg/kg dolutegravir (DTG)^57^. At 2, 6, and 10 weeks post-ART initiation, RMs were administered intramuscularly either 300 µg each of HIV b.63521 (B.63521_mutC)^58^ + 1086.C gp120 (C.1086_D7gp120K160N/293 F)^59,60^ protein (*n* = 6) or 50 µg RSV (*n* = 6) immunization with STR8S-C adjuvant (squalene-containing STS based adjuvant plus R848 plus CpG oligodeoxynucleotides)^58,61^. ART was discontinued at 24 wpi, and RMs were monitored for an additional 8 weeks.

### Viral RNA load quantification

Plasma RNA load was quantified using a well-established quantitative reverse transcriptase (RT) PCR assay targeting SIVgag RNA, as previously described^56^. RNA was isolated from plasma samples using the QIAsymphony Virus/Bacteria Midi kit on the QIAsymphony SP automated sample preparation platform (Qiagen, Hilden, Germany). RNA was extracted manually if plasma volumes were limited. Data reported are the number of SIV RNA copy equivalents per ml of plasma, with a limit of detection of 15 copies/ ml. Upon cessation of ART, viral rebound was defined as HIV RNA levels of >150 copies/mL (10x detection limit of the assay).

### Recombinant protein and soluble CD4 blocking ELISA

Env-specific IgG binding was assessed in plasma in a 384-well plate format. The plates were coated overnight with HIV CH505, b.65321, or 1086.C gp120 or V3 antigens (30 ng/well) and then blocked with the assay diluent (phosphate-buffered saline containing 4% whey, 15% normal goat serum, and 0.5% Tween-20). Dilutions of plasma were then added to the plates and incubated for 1 h, followed by detection with a horseradish peroxidase (HRP)-conjugated antibody, polyclonal goat anti-monkey IgG (1:4000; Rockland, Gilbertsville, PA, Cat# 617-103-130). The plates were developed by using the ABTS-2 peroxidase substrate system (KPL, Gaithersburg, MD). Macaca mulatta purified IgG (NIH Nonhuman Primate Reagent Resource, R24 OD010976, U24 AI126683) was used to develop standard curves (1000 ng/ml for 12-wells in 2-fold series), and the concentration of IgG antibody was calculated relative to the standard using a 5-parameter fit curve (SoftMax Pro 7; Molecular Devices, San Jose, CA). For monoclonal antibodies, effective concentration 50% (EC50) was calculated by the concentration of antibody which resulted in a 50% reduction in optical density (OD) from the maximum value.

For CD4 blocking ELISAs, 384-well plates (Corning Life Sciences, Lowell, MA) were coated with 1086.C gp120 at 30 ng/well. Following the same steps as previously stated, the plates were blocked with assay diluent and serial dilutions of monoclonal antibody and plasma were distributed to the plates. Once the mAbs and the plasma were added and incubated for 1 h, soluble CD4 (sCD4) (NIH AIDS Reagent Program, Division of AIDS, NIAID, NIH: Human Soluble CD4 Recombinant Protein (sCD4) from Progenics, Cat# 4615) was added at 64 µg/mL. The sCD4 binding was detected using a biotinylated Human anti-CD4 (Thermo Fisher Scientific, San Diego, CA, Cat# 13-0048-82) at 0.09 µg/mL followed by HRP-conjugated Streptavidin (1:10,000; Thermo Fisher Scientific, San Diego, CA, Cat# N100). Percent sCD4 binding inhibition was calculated as follows: 100 – (average of sera duplicate OD/average of negative control OD) x 100. OD referring to optical density. A reduction of absorbance by >50% by vaccine-elicited Abs present in plasma indicated blocking of sCD4 binding to 1086.C gp120.

### Binding Antibody Multiplex Assays (BAMA)

HIV-1 antigens (50 ug) were covalently coupled to carboxylated polystyrene beads (Bio-Rad, Hercules, CA)^62^, then binding of IgG to the bead-conjugated HIV-1 Env antigens was measured in plasma samples from the treated and control groups. The positive control was purified IgG from a pool of plasma of HIV vaccinated rhesus macaques (RIVIG). The conjugated beads were incubated on filter plates (Millipore, Stafford, VA) for ~30 min before plasma samples were added. The plasma samples were diluted in assay diluent (1% dry milk + 5% goat serum + 0.05% Tween-20 in 1X phosphate buffer saline, pH 7.4.) at 1:50 (panel 1) or 1:400 (panel 2) and incubated with conjugated beads for 30 min. IgG binding was detected using a PE-conjugated mouse anti-monkey IgG (Southern Biotech, Birmingham, Alabama, Cat# 4700-09) at 4 μg/mL. The beads were washed and acquired on a Bio-Plex 200 instrument (Bio-Rad, Hercules, CA) and IgG binding was expressed as mean fluorescence intensity (MFI). To assess assay background, the MFI of binding to wells that did not contain beads or sample (blank wells) and non-specific binding of the samples to unconjugated blank beads were evaluated during assay analysis. High background detection for plasma samples were noted and repeated if necessary. An HIV-envelope specific antibody response was considered positive if above the lower limit of detection (100 MFI). To check for consistency between assays, the EC50 and maximum MFI values of the positive control (RIVIG) was tracked by Levy-Jennings charts. The antigens conjugated to the polystyrene beads were run in two panels. Panel 1 included the following antigens:1086 C K160N V1V2, b.63521 v1v2, b.63521 v2, SHIV.C.CH505.TF V2, CH505.TF V1V2; panel 2: 1086 C K160N gp120, CH505.TF gp120, 1086.C gp140, CH505.TF gp140, A1.Con_env03 gp140, A244 gp120, B.Con_env03 gp140, Con6 gp120, ConC gp120, MN gp120, Bio.V3.C, gp70 MNV3, SHIV.CH505 V3, 1086 C V3, b.63521 V3, Bio RV144 C5.2.C, C1.

#### Surface Plasmon Resonance (SPR) measurements of purified plasma IgG avidity

IgG avidity to a panel of HIV-1 antigens (CH505TF gp120 & C.1086 gp120K160N) was measured by surface plasmon resonance (BIAcoreTM 3000, BIAcore/GE Healthcare, Pittsburgh, PA) analysis. Using a multiplex array format (2 × 2), binding response was measured by SPR following immobilization by amine coupling of envelope protein^63^ on CM5 sensor chips (BIAcore/GE Healthcare, Pittsburgh, PA). Purified plasma IgG samples at 200 µg/ml were flowed (2.5 min) over spots (chip surfaces) of antigen followed by a dissociation phase (post-injection/buffer wash) of 10 min and a regeneration with Glycine pH2.0. Non-specific IgG binding of a pre-immune sample was subtracted from each post-immunization IgG sample binding data. Data analyses were performed with BIA-evaluation 4.1 software (BIAcore/GE Healthcare, Pittsburgh, PA). Binding responses were measured by averaging post-injection response unit (RU) over a 10 s window; and dissociation rate constant, kd (second-1), was measured during the post-injection phase after stabilization of signal. Positive response was defined when both replicates have a RU value ≥10. Relative avidity binding score is calculated as follows: Avidity score (RU.s) = (Binding Response Units /kd)^64^.

### Neutralization assays

Neutralization by antibodies in plasma of MW965.LucR.T2A.ecto/293 T IMC (clade C, tier 1), CH505 w4.3 HIV-1 pseudovirus (clade C, tier 1a), and autologous CH505.TF (clade C, tier 2) HIV-1 pseudovirus was measured in TZM-bl cells (NIH AIDS Reagent Program, Division of AIDS, NIAID, NIH; from John Kappes) via a reduction in luciferase reporter gene expression after a single round of infection^65–67^. Prior to screening, plasma was heated-inactivated at 56 °C for 30 min. Serially diluted plasma (eight dilutions, threefold stepwise) was incubated with HIV-1 pseudovirus in duplicate for 1 h at 37 °C in a total volume of 150 μl growth medium (DMEM + 10% FBS + 2% + 25 mM HEPES + 1X Pen-Strep) in 96-well flat-bottom culture plates (Corning-Costar). Freshly trypsinized TZM-bl cells (10,000 cells in 100 μl of growth medium containing 75 μg/ml DEAE-dextran) were then added to each well and incubated for 48 h. After 48 h, 150 μl of culture medium was removed from each well and 100 μl of Bright Glo reagent (Promega, used as per manufacturer’s recommendation) was added to the cells. After a 2 min incubation at room temperature to allow cell lysis, 150 μl was transferred to 96-well black solid plates for measurements of luminescence. Luminescence was measured using a Victor X3 multilabel plate reader, 1 s per well (PerkinElmer). The ID_50_ was calculated as the dilution that resulted in a 50% reduction in relative luminescence units (RLU) compared to virus control wells. The monoclonal antibody, b12R1, was used as a positive control for MW965 assays, and VRC01 was used a positive control for all other assays at 22.2 μg/ml starting concentration in 3-fold serial dilution.

### ADCC

The ADCC-GTL assay was used to measure plasma ADCC activity^68^. Briefly, CEM.NKRCCR5 target cells (NIH AIDS Reagent Program, Division of AIDS, NIAID, NIH; from Alexandra Trkola)^69^ were coated with recombinant 1086.C or CH505 gp120. Cryopreserved human peripheral blood mononuclear cells (PBMCs) from an HIV-1 seronegative donor with the heterozygous 158 F/V genotype for the Fcγ receptor IIIa were used as the source of effector cells^70,71^. Plasma samples were tested after a 4-fold serial dilution starting at 1:100. ADCC was measured as percent Granzyme B (GzB) activity, defined as the frequency of target cells positive for proteolytically active GzB out of the total viable target cell population. Final results are expressed after subtraction of the background GzB activity observed in wells containing target and effector cells in the absence of plasma. ADCC endpoint titers were determined by interpolating the last positive dilution of plasma (>8% GzB activity).

### Plasma binding to the surface of HIV-1-infected cells

Indirect surface staining was used to measure the ability of plasma samples to bind HIV-1 envelope expressed on the surface of infected cells^72^. CEM.NKR_CCR5_ cells were mock infected or infected with HIV-1 infectious molecular clones^73^ expressing the 1086.C or CH505.TF envelope proteins. The cells were incubated with a 1:100 dilution of plasma samples for 2 h at 37 °C and then stained with Live/Dead Aqua Dead Cell Stain (Thermo, Fisher Scientific, Waltham, MA) to exclude dead cells from analysis. Cells were washed and then permeabilized with Cytofix/Cytoperm solution (BD Biosciences, San Jose, CA) prior to staining with fluorescein isothiocyanate (FITC)-conjugated goat anti-rhesus IgG(H + L) polyclonal antiserum (Southern Biotech, Birmingham, AL, Cat# 6200-02) and RD1-conjugated anti-p24 MAb KC57 (Beckman Coulter, Inc., Indianapolis, IN, Cat# 6604667). Cells positive for plasma binding were defined as viable, p24 positive, and FITC positive. Final results are reported as the FITC MFI of the live infected cell population (p24-positive cells) after subtraction of the background observed for the pre-infection (week 0) samples.

### HIV envelope-specific memory B cell phenotyping

For phenotyping of CH505 and 1086.C gp120-specific memory B cells, suspension of 3 × 10^6^ PBMCs were blocked with 6.25 μg/ml anti-human CD4 antibody (BD Biosciences) at 4 °C for 15 min. After incubation, PBMCs were washed twice with PBS, and pelleted at 300 g for 5 min. Following incubation and wash with PBS, PBMCs were then stained with a cocktail of fluorescently conjugated antibodies for surface markers including CD20, CD3, IgM, CD16, CD8, IgD PE, CD14 and CD27 (Supplementary Table 2) and custom-conjugated BV421-HIV-1 gp120 (SHIV.C.CH505 T/F) and AF647-HIV-1 gp120 (1086.C K160N) prepared as described previously^74^. PBMCs were then incubated at 4 °C with LIVE/DEAD Fixable Aqua Dead Cell Stain Kit (Thermo Fisher Scientific) for 30 min. The stained PBMCs were acquired on an LSRII flow cytometer (BD Biosciences) using BD FACS Diva software and analyzed with FlowJo software version 10. The following gating strategy was applied: lymphocytes were gated on singlets and live cells were selected to gate on CD14^−^CD16^-^ cells (monocytes/macrophages) and CD20^+^cells (B cells). B cells were further gated on IgD^-^ memory B cells (Supplementary Fig. 1). Frequencies of B cells positive for both BV421- CH505 gp120 and AF647-1086.C gp120, and each gp120 alone are reported. FMOs for each gp120 conjugated antigen were used to define positive threshold for cell populations. PBMCs from 3 RMs from a pre-infection time point (week 0) were also included in the analysis as an additional control. For a detailed list of antibodies used for B cell phenotyping, see Supplementary Table 2.

### Single genome amplification (SGA) of HIV-1 env gene

Viral RNA was purified from the plasma sample for each animal using the EZ1 Viral RNA Mini Kit 2.0 (Qiagen 955134) and transcribed to cDNA using 1X reaction buffer, 0.5 mM of each deoxynucleoside triphosphate (dNTP), 5 mM DDT, 2 U/mL RNaseOUT, 10 U/mL of SuperScript III reverse transcription mix (Invitrogen), and 0.25 mM antisense primer SHIVEnv.R3out (5'-CTAATTCCTGGTCCTGAGGTGTAATCCTG-3'). The cDNA was end-point diluted in 96-well plates (Applied Biosystems, Inc.) and PCR amplified with the Platinum Taq High Fidelity Kit (Invitrogen) to get a positive PCR product yield of <30% to maximize the likelihood of amplification from a single genome. Two rounds of PCR amplification were conducted for each dilution with 2 µL of the first-round PCR products used as the template for the second-round PCR. SIVmac.F4out (5'-TCATATCTATAATAGACATGGAGACACCC-3') and SHIVEnvR3out (5'- CTAATTCCTGGTCCTGAGGTGTAATCCTG-3') were used as the primer pair in round 1. SIVmac766.F2in (5'- GGAAATCCTCTCTCAACTATACCGCCCTC -3') and SIVmac766.R2in (5'- CTATTGCCAATTTGTAACTCATTGTTC-3') were used as the primer pair in round 2. PCR was carried out using 1X buffer, 2 mM MgSO_4_, 0.2 mM of each dNTP, 0.2 μM of each primer, and 0.025 U/µL Platinum Taq High Fidelity polymerase (Invitrogen) in a 20 µL reaction in round 1 and in a 50 µL reaction in round 2. Round 1 and round 2 amplification conditions were 1 cycle of 94 °C for 2 min; 35 cycles of 94 °C for 15 s, 55 °C for 30 s, and 68 °C for 3 min and 30 s; followed by 1 cycle of 68 °C for 10 min. Round 2 PCR amplicons were visualized by agarose gel electrophoresis, purified using the Agencourt AMPure XP Magnetic Bead PCR Purification system, and sent to Genewiz, Inc. or the DHVI Viral Genome Analysis core for sequencing using an ABI3730xl genetic analyzer (Applied Biosystems).

Partially overlapping sequences from each amplicon were assembled and edited using Sequencher (Gene Codes, Inc). Sequences with double peaks per base read were discarded. Sequences with only a single double peak were retained as that most likely represented a Taq polymerase error in an early round of PCR rather than multiple template amplification. These sequence ambiguities were read as the consensus nucleotide from the other overlapping sequences. Full-length envelope sequences were aligned and edited using Seaview Version 4^75^. Neighbor-joining phylogenetic trees were generated using FigTree and MEGAX^76^. Highlighter mutations plots were created using a highlighter tool freely available from the Los Alamos National Laboratory HIV Sequence Database: https://www.hiv.lanl.gov/content/sequence/HIGHLIGHT/highlighter_top.html.

### Statistical methods

Time to viral rebound was compared between the vaccinated and placebo animals using a log-rank test with exact *p*-value. The 4 animals that never experienced rebound were right-censored at 32 wpi (8 weeks after discontinuation of ART). Primary endpoint analysis utilized Wilcoxon signed-rank tests with exact *p*-values to compare vaccine (1086c and b.63521) versus challenge virus antigen (CH505) specific responses among HIV Env vaccinated RMs (*n* = 6). To adjust for multiple comparisons, the Benjamini–Hochberg (BH) procedure was used to control the false discovery rate (FDR). Adjustments to control the FDR at *α* = 0.05 were performed for the pre-specified primary endpoints for a total of 13 tests (Supplementary Table 5). Secondary endpoint analysis compared immune responses of primary interests between HIV Env vaccinated RMs and placebo vaccinated RMs at specific time points over the course of the study. Wilcoxon rank-sum tests with exact *p*-values, adjusted to control the FDR at *α* = 0.05 as described above, were calculated for the pre-specified secondary endpoints for a total of 44 tests (Supplementary Table 3). The set of statistical analyses defined as exploratory endpoints compared a subset of immune responses between the two vaccine groups. Wilcoxon rank-sum tests with exact *p*-values, adjusted to control the FDR at *α* = 0.05 as described above, were calculated for the pre-specified secondary endpoints for a total of 14 tests (Supplementary Table 4). Both the unadjusted (raw_p) and FDR-adjusted (FDR_p) *p*-values are reported in Supplementary Tables 3–5. All statistical tests were performed using SAS version 9.4 (Cary, NC, USA). Spearman’s rank-order correlation was estimated between time to viral rebound and assay values, including viral load at week 12, ADCC antibody titers (weeks 10, 20, and 28), CH505TF virus neutralization (weeks 12, 26, and 28), and avidity score (weeks 12 and 20; Supplementary Fig. 5). Subjects with no viral rebound observed were imputed to the highest rank by assigning a number greater than the maximum observed rebound time, and assay values below the limit of detection were imputed to the lowest rank by assigning a value at half of the assay’s lower limit of detection for correlation estimates.

### Reporting summary

Further information on research design is available in the Nature Research Reporting Summary linked to this article.

## Supplementary information

Supplemental Material

REPORTING SUMMARY

## Data Availability

The datasets generated and/or analyzed during the current study are available from the corresponding author on reasonable request. All sequences have been deposited in GenBank with accession numbers: OM686910-OM686980 for pre-ART Env sequences, and OM906830-OM906850 for post-ART sequences.
