# Supplementary material for: Leveraging antigenic seniority for maternal vaccination to prevent mother-to-child transmission of HIV-1
Source: NPJ Vaccines. 2022 Jul 30;7:87. doi: 10.1038/s41541-022-00505-w (PMC9338948; doi:10.1038/s41541-022-00505-w)
Supplement: Supplementary file 1 — Supplemental Material [file 41541_2022_505_MOESM1_ESM.pdf]

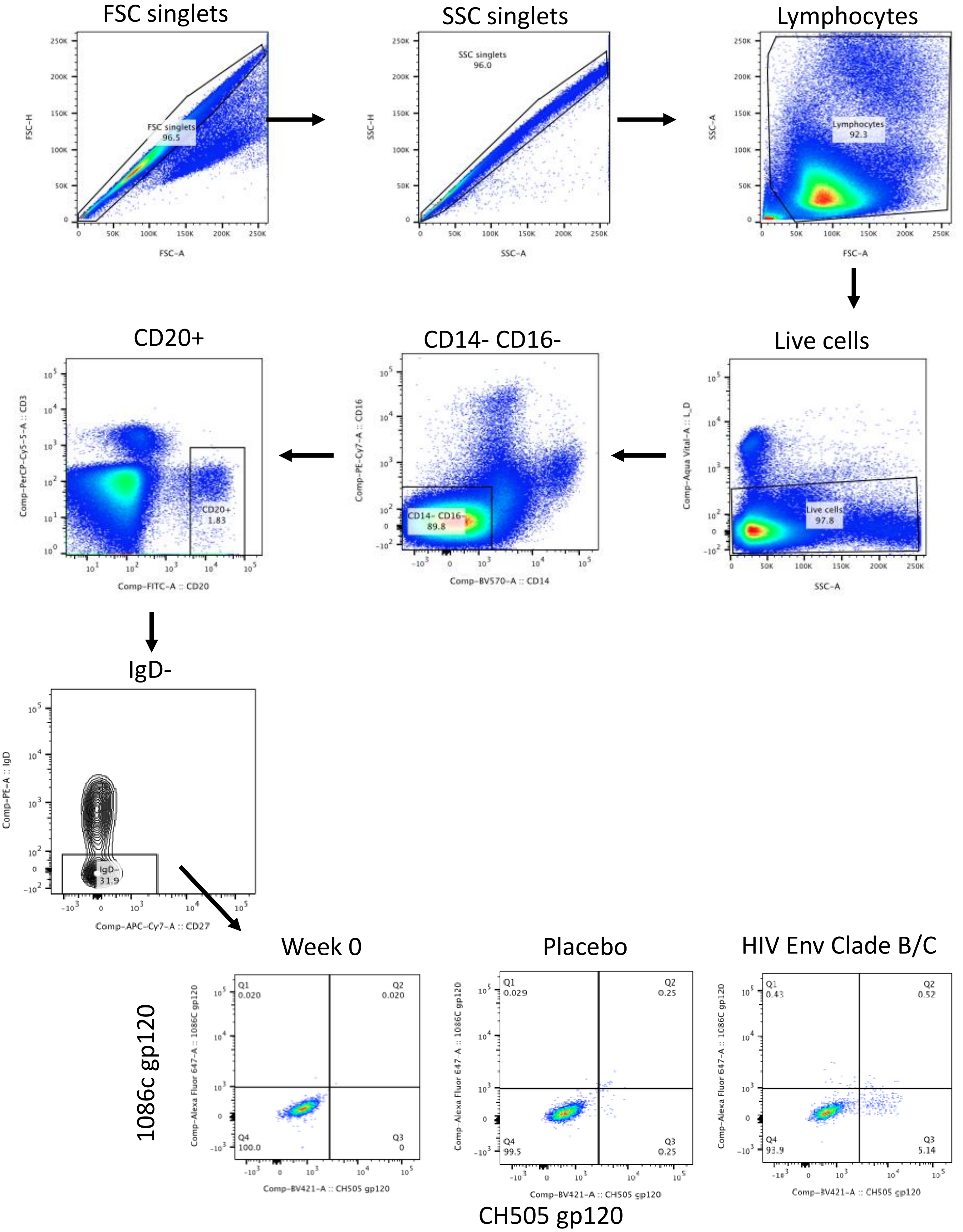

Supplementary Figure 1. Gating strategy used to identify CH505 and 1086c gp120-specific memory B cells. Plots are representative of one RM from each cohort.

Supplementary Table 1. SGA summary table. Time points and viral loads at which pre- and +t-ART/immunization samples for each animal were analyzed are shown along with the number of SGAs that have been successfully isolated.

Pre-ART/Immunization	PTID	Weeks Post-Infection	Viral Load (vRNA copies/mL)	SGAs
HIV Clade B/C	42814	10	2900	12
	43633	8	1100	25
Placebo	43268	8	52000	9
	39950	12	28000	29
**Rebound**		**Weeks Post-ART**	**Viral Load (vRNA copies/mL)**	**SGAs**
HIV Clade B/C	42814	3 (27wpi)	3700	1
	43633	4 (28 wpi)	1800	4
Placebo	43268	3 (27wpi)	1500	6
	39950	2 (27 wpi)	13000	14

a. Placebo Phylogenetic Trees

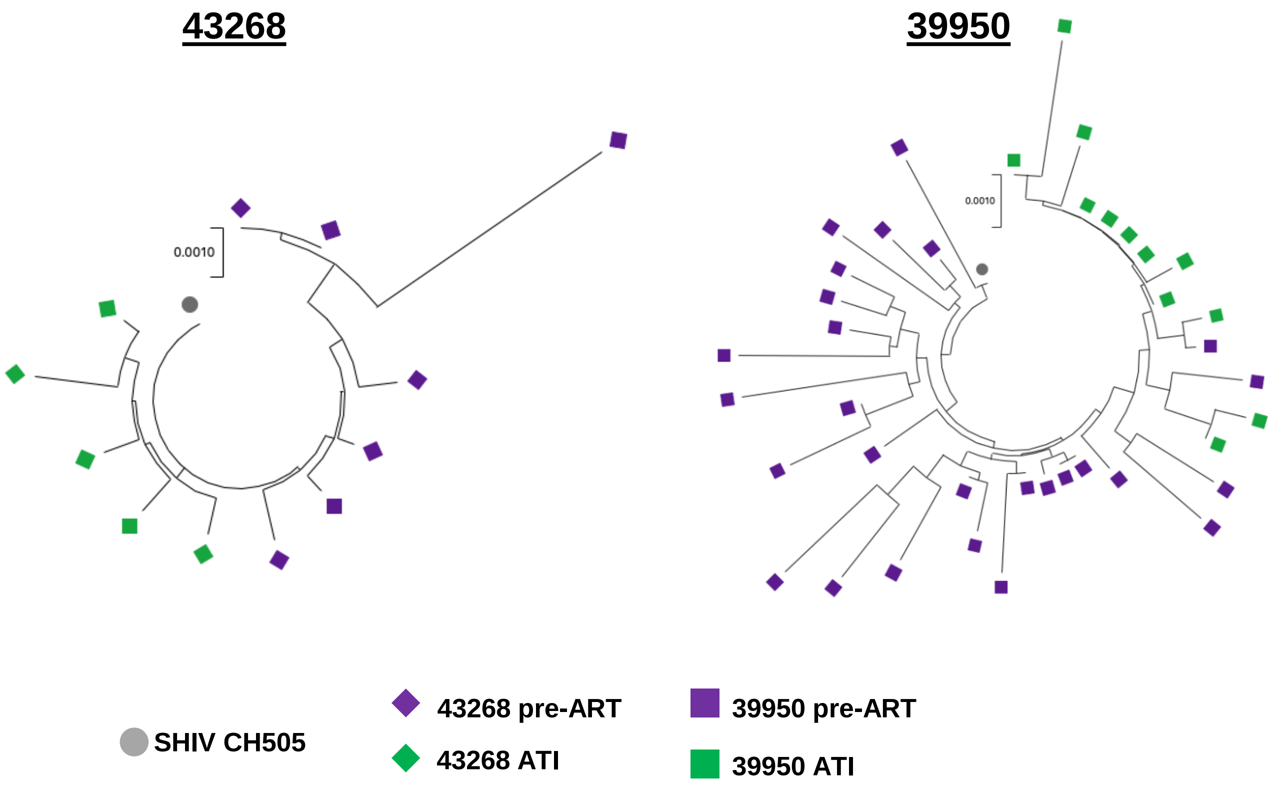

b. HIV Env Clade B/C Phylogenetic Trees

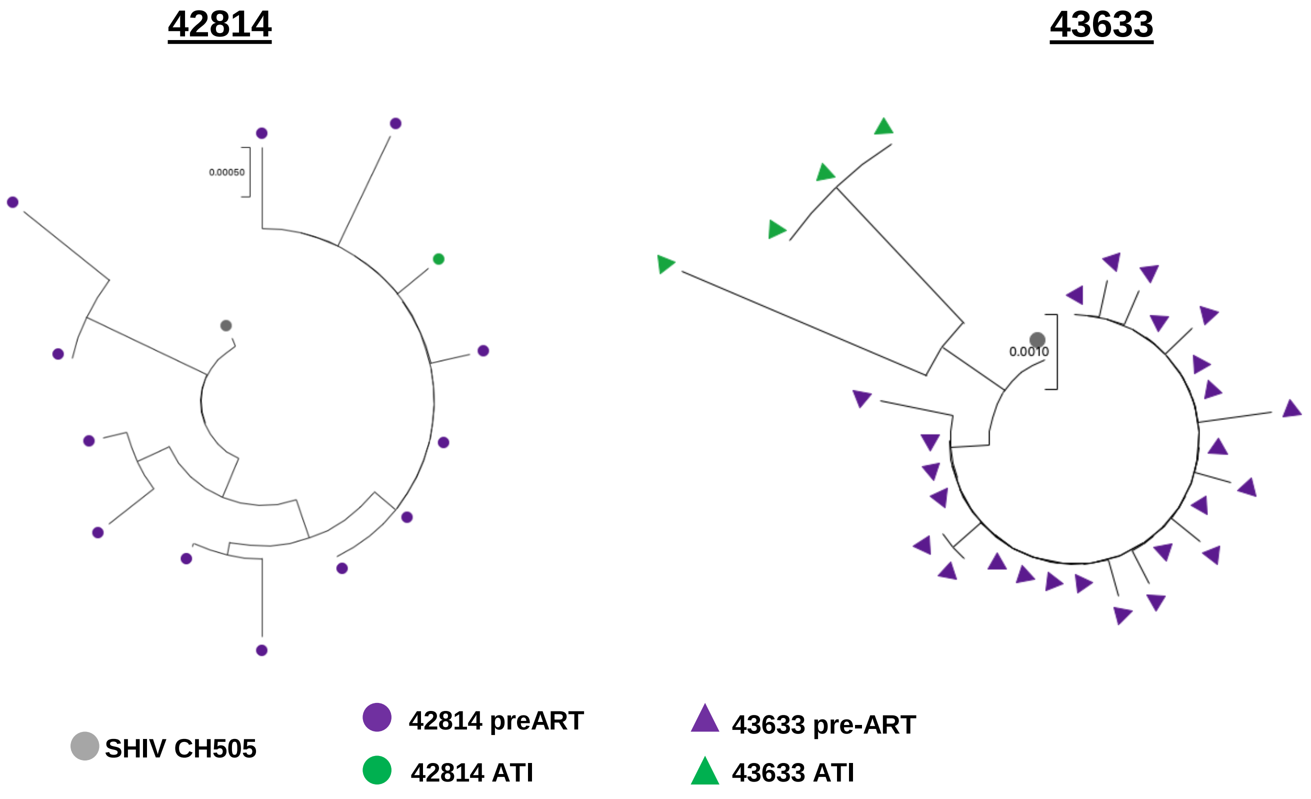

Supplementary Figure 2. **Neighbor-joining phylogenetic tree of pre-ART and ATI Env sequences from vaccinated and placebo RMs.** Phylogenetic trees were constructed for Placebo (A) and HIV Env vaccinated (B) RMs for nucleotide sequences of *env* gene prior to treatment initiation (purple), and during ATI (green) using the time-point of peak viral load during rebound . Each tree is rooted to the SHIV.C.CH505 challenge virus (gray).

Pre-ART/Immunization Env Sequence Comparison

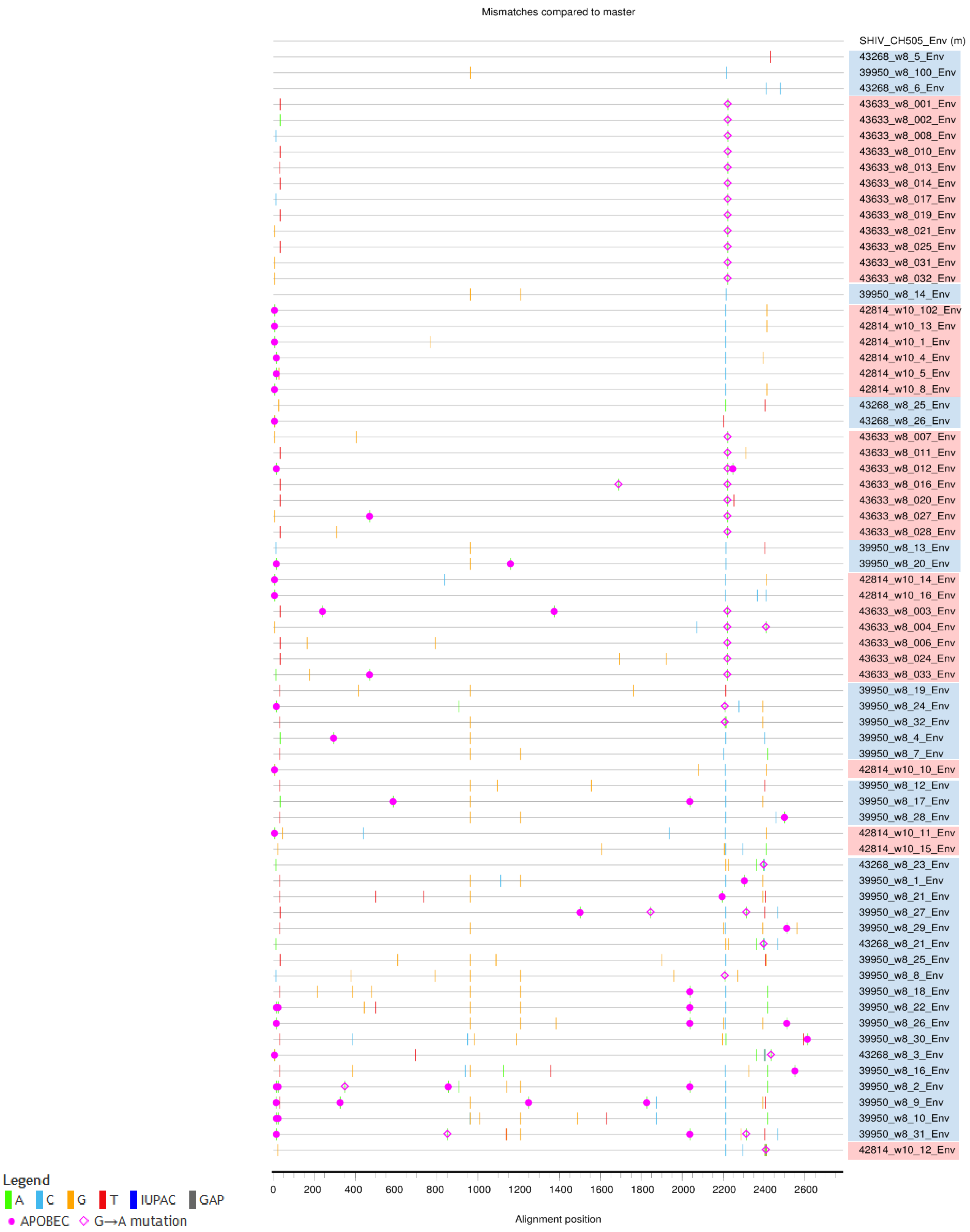

Supplementary Figure 3. Highlighter plot of HIV Env diversity pre-ART from vaccinated and placebo RMs. Highlighter plot comparing pre-ART/immunization (A) *env* sequences to the SHIV.C.CH505 challenge virus master *env* sequence +itioned at the top of the plot. Each colored tick mark is a mutation compared to the master sequence. HIV Env vaccinated animals are highlighted in red, and placebo recipients are highlighted in blue.

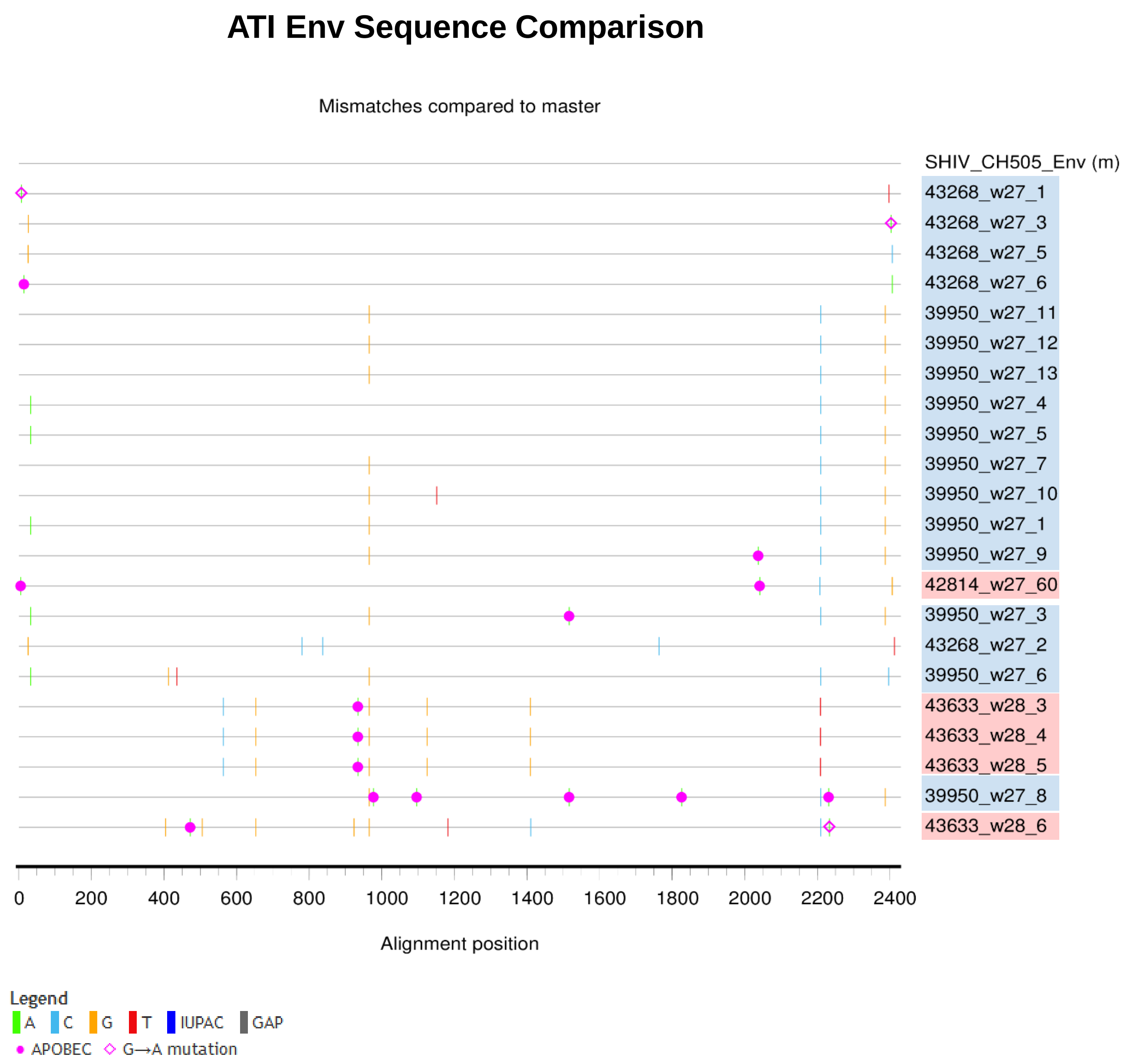

Supplementary Figure 4. Highlighter plot of diversity among ATI *env* sequences from vaccinated and placebo RMs. Highlighter plot comparing rebound *env* sequences to the SHIV.C.CH505 challenge virus master *env* sequence positioned at the top of the plot. Each colored tick mark is a mutation compared to the master sequence. HIV Env vaccinated animals are highlighted in red, and placebo recipients are highlighted in blue.

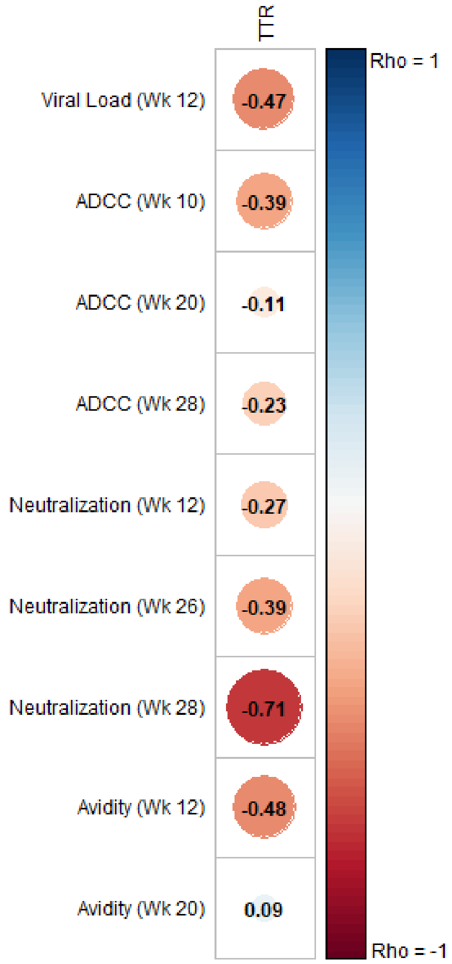

Supplementary Figure 5. Correlogram showing spearman correlations among viral load at week 12, CH505-specific ADCC antibody titers, CH505TF virus neutralization, and CH505-specific avidity scores. The size of the circle and the color (blue is a positive correlation, red is negative) indicate the strength and direction of the association, and the estimated Spearman correlation coefficient is displayed in the center.

Supplementary Table 2. Antibodies used in flow cytometry analysis. Table shows the list of each antibody used in flow cytometry for HIV Env-specific B cell phenotyping including the clone of each mAb used and the manufacturer.

Marker	Fluorophore	Clone	Manufacturer	Catalogue #	Experiment	Staining
CD20	FITC	L27	BD Biosciences	347673	Env-specific B cell phenotyping	surface
CD3	PerCP Cy5.5	SP34-2	BD Biosciences	552852	Env-specific B cell phenotyping	surface
IgD	PE	polyclonal	Southern Biotech	2030-09	Env-specific B cell phenotyping	surface
CD8	PE Texas Red	3B5	Invitrogen	MHCD0817	Env-specific B cell phenotyping	surface
IgM	PE Cy5	G20-127	BD Biosciences	551079	Env-specific B cell phenotyping	surface
CD16	PE Cy7	3G8	BD Biosciences	557744	Env-specific B cell phenotyping	surface
CD14	BV570	M5E2	Biolegend	301832	Env-specific B cell phenotyping	surface
CD27	APC Cy7	O323	Biolegend	302816	Env-specific B cell phenotyping	surface

Supplementary Table 3. Secondary endpoint analysis comparing immune responses of primary interests between HIV Env vaccinated and placebo vaccinated RMs at specific time points. Wilcoxon rank-sum tests with exact p-values, adjusted to control the FDR at α = 0.05. FDR-adjusted p-values <0.05 are in bold.

Assay	Outcome	Antigen/Pseudovirus	Weeks post-infection	Raw_P	fdr_p
Elisa	Concentration binding	ch505tf_gp120_293f	12	0.937	1.000
Elisa	Concentration binding	ch505tf_gp120_293f	16	0.002	**0.005**
Elisa	Concentration binding	ch505tf_gp120_293f	20	0.002	**0.005**
Elisa	Concentration binding	ch505tf_gp120_293f	24	0.002	**0.005**
Elisa	Concentration binding	1086d7_gp120k160n	12	0.699	0.961
Elisa	Concentration binding	1086d7_gp120k160n	16	0.002	**0.005**
Elisa	Concentration binding	1086d7_gp120k160n	20	0.002	**0.005**
Elisa	Concentration binding	1086d7_gp120k160n	24	0.002	**0.005**
Elisa	Concentration binding	b_63521_d11gp120	12	0.699	0.961
Elisa	Concentration binding	b_63521_d11gp120	16	0.002	**0.005**
Elisa	Concentration binding	b_63521_d11gp120	20	0.002	**0.005**
Elisa	Concentration binding	b_63521_d11gp120	24	0.002	**0.005**
Elisa	Concentration binding	CH505_tf_v3	12	0.937	1.000
Elisa	Concentration binding	CH505_tf_v3	16	0.002	**0.005**
Elisa	Concentration binding	CH505_tf_v3	20	0.002	**0.005**
Elisa	Concentration binding	CH505_tf_v3	22	0.002	**0.005**
Elisa	Concentration binding	1086c_k160n_v3	12	0.589	0.863
Elisa	Concentration binding	1086c_k160n_v3	16	0.002	**0.005**
Elisa	Concentration binding	1086c_k160n_v3	20	0.002	**0.005**
Elisa	Concentration binding	1086c_k160n_v3	22	0.002	**0.005**
Elisa	Concentration binding	b_63521_v3	12	0.937	1.000
Elisa	Concentration binding	b_63521_v3	16	0.002	**0.005**
Elisa	Concentration binding	b_63521_v3	20	0.002	**0.005**
Elisa	Concentration binding	b_63521_v3	22	0.002	**0.005**
ADCC	Endpoint titer_dilution	1086d7_gp120k160n	10	0.792	1.000
ADCC	Endpoint titer_dilution	1086d7_gp120k160n	14	0.234	0.367
ADCC	Endpoint titer_dilution	1086d7_gp120k160n	20	0.002	**0.005**
ADCC	Endpoint titer_dilution	1086d7_gp120k160n	28	0.024	**0.046**
ADCC	Endpoint titer_dilution	1086d7_gp120k160n	32	0.065	0.110
ADCC	Endpoint titer_dilution	ch505tf_gp120_293f	10	0.931	1.000
ADCC	Endpoint titer_dilution	ch505tf_gp120_293f	14	1.000	1.000
ADCC	Endpoint titer_dilution	ch505tf_gp120_293f	20	0.002	**0.005**
ADCC	Endpoint titer_dilution	ch505tf_gp120_293f	28	0.002	**0.005**
ADCC	Endpoint titer_dilution	ch505tf_gp120_293f	32	0.065	0.110
Neutralization	ID50	CH0505TF	12	0.922	1.000
Neutralization	ID50	CH0505TF	26	1.000	1.000
Neutralization	ID50	CH0505TF	28	1.000	1.000
Neutralization	ID50	CH0505TF	32	1.000	1.000
Neutralization	ID50	CH0505.w4.3	12	0.387	0.588
Neutralization	ID50	CH0505.w4.3	26	0.061	0.110
Neutralization	ID50	CH0505.w4.3	28	0.981	1.000
Neutralization	ID50	MW965.26.LucR.T2A.ecto	12	0.937	1.000
Neutralization	ID50	MW965.26.LucR.T2A.ecto	26	0.004	**0.009**
Neutralization	ID50	MW965.26.LucR.T2A.ecto	28	0.180	0.293

Supplementary Table 4. Exploratory endpoints comparing a subset of immune responses between the two vaccine groups. Wilcoxon rank-sum tests with exact p-values, adjusted to control the FDR at α = 0.05

Assay	Outcome	Antigen	Weeks post-infection	Raw_P	fdr_p
B cell phenotyping	CH505_gp120_sp	CH505TF_D8gp120/293F	24	0.008	0.061
B cell phenotyping	1086c_gp120_sp	C.1086_D7gp120K160N/293F	24	0.016	0.074
Avidity	ant1_avidity score	CH505TF_D8gp120/293F	12	0.937	0.937
Avidity	ant1_avidity score	CH505TF_D8gp120/293F	20	0.065	0.130
Avidity	ant2_avidity score	C.1086_D7gp120K160N/293F	12	0.699	0.753
Avidity	ant2_avidity score	C.1086_D7gp120K160N/293F	20	0.026	0.091
Avidity	ant3_avidity score	B.63521_D11gp120_mutC/293F	12	0.662	0.753
Avidity	ant3_avidity score	B.63521_D11gp120_mutC/293F	20	0.065	0.130

Supplementary Table 5. Primary endpoint analysis comparing vaccine (1086c and b.63521) versus challenge virus antigen (CH505) specific responses among HIV Env vaccinated RMs. Wilcoxon signed-rank tests with exact p-values , adjustments to control the FDR at α = 0.05.

Assay	Antigen 1	Antigen 2	Weeks post-infection	raw_p	fdr_p
Elisa	CH505	1086c	12	0.031	0.058
Elisa	CH505	1086c	16	0.031	0.058
Elisa	CH505	1086c	20	0.031	0.058
Elisa	CH505	1086c	24	0.063	0.074
Elisa	CH505	B63521	12	0.031	0.058
Elisa	CH505	B63521	16	0.031	0.058
Elisa	CH505	B63521	20	0.031	0.058
Elisa	CH505	B63521	24	0.031	0.058
Avidity	CH505	1086c	12	0.063	0.074
Avidity	CH505	1086c	20	0.313	0.313
Avidity	CH505	B63521	12	0.063	0.074
Avidity	CH505	B63521	20	0.156	0.169
B cell phenotyping	CH505	1086c	24	0.063	0.074
