# Supplementary material for: Leveraging antigenic seniority for maternal vaccination to prevent mother-to-child transmission of HIV-1
Source: NPJ Vaccines. 2022 Jul 30;7:87. doi: 10.1038/s41541-022-00505-w (PMC9338948; doi:10.1038/s41541-022-00505-w)
Supplement: Supplementary file 2 — REPORTING SUMMARY [file 41541_2022_505_MOESM2_ESM.pdf]

nature portfolio

Corresponding author(s): Sallie R. Permar

Last updated by author(s): Mar 8, 2022

Reporting Summary

Nature Portfolio wishes to improve the reproducibility of the work that we publish. This form provides structure for consistency and transparency in reporting. For further information on Nature Portfolio policies, see our Editorial Policies and the Editorial Policy Checklist.

Statistics

For all statistical analyses, confirm that the following items are present in the figure legend, table legend, main text, or Methods section.

n/a	Confirmed	
☐	☒	The exact sample size (*n*) for each experimental group/condition, given as a discrete number and unit of measurement
☐	☒	A statement on whether measurements were taken from distinct samples or whether the same sample was measured repeatedly
☐	☒	The statistical test(s) used AND whether they are one- or two-sided<br>*Only common tests should be described solely by name; describe more complex techniques in the Methods section.*
☐	☒	A description of all covariates tested
☐	☒	A description of any assumptions or corrections, such as tests of normality and adjustment for multiple comparisons
☐	☒	A full description of the statistical parameters including central tendency (e.g. means) or other basic estimates (e.g. regression coefficient) AND variation (e.g. standard deviation) or associated estimates of uncertainty (e.g. confidence intervals)
☐	☒	For null hypothesis testing, the test statistic (e.g. *F*, *t*, *r*) with confidence intervals, effect sizes, degrees of freedom and *P* value noted<br>*Give P values as exact values whenever suitable.*
☒	☐	For Bayesian analysis, information on the choice of priors and Markov chain Monte Carlo settings
☒	☐	For hierarchical and complex designs, identification of the appropriate level for tests and full reporting of outcomes
☐	☒	Estimates of effect sizes (e.g. Cohen's *d*, Pearson's *r*), indicating how they were calculated

Our web collection on statistics for biologists contains articles on many of the points above.

Software and code

Policy information about availability of computer code

Data collection	No software was used to collect data.
Data analysis	SoftMax Pro 7, BIA-evaluation 4.1 software, FlowJo software version 10, FigTree v4.0 and MEGAX, All statistical tests were performed using SAS version 9.4

For manuscripts utilizing custom algorithms or software that are central to the research but not yet described in published literature, software must be made available to editors and reviewers. We strongly encourage code deposition in a community repository (e.g. GitHub). See the Nature Portfolio guidelines for submitting code & software for further information.

Data

Policy information about availability of data

All manuscripts must include a data availability statement. This statement should provide the following information, where applicable:

- Accession codes, unique identifiers, or web links for publicly available datasets
- A description of any restrictions on data availability
- For clinical datasets or third party data, please ensure that the statement adheres to our policy

The datasets generated and/or analyzed during the current study are available from the corresponding author on reasonable request. All sequences have been deposited in GenBank with accession numbers: OM686910-OM686980 for pre-ART Env sequences, and OM906830-OM906850 for post-ART sequences.

March 2021

Field-specific reporting

Please select the one below that is the best fit for your research. If you are not sure, read the appropriate sections before making your selection.

☒ Life sciences ☐ Behavioural & social sciences ☐ Ecological, evolutionary & environmental sciences

For a reference copy of the document with all sections, see nature.com/documents/nr-reporting-summary-flat.pdf

Life sciences study design

All studies must disclose on these points even when the disclosure is negative.

Sample size	For this proof-of-concept study the samples size was n=6 per group (vaccinated vs. placebo). Based on our previous experiences with NHP models of HIV Env vaccination, this sample size can differentiate large differences between treatment groups.
Data exclusions	No data was excluded from analyses.
Replication	Samples for immunological assays were run in duplicates. Multiple time points were assessed where applicable.
Randomization	Twelve female, adult rhesus macaques (RMs) were infected intravenously with SHIV.C.CH505, and started on a daily ART regimen at 12 weeks post-infection. Prior to vaccination, two groups of six were balanced based on plasma viral load at 12 wpi. Six RMs were assigned to the placebo vaccine group, and the remaining six received an HIV Env combined Clade B/C vaccine.
Blinding	Immunological and virological assays were performed blinded.

Reporting for specific materials, systems and methods

We require information from authors about some types of materials, experimental systems and methods used in many studies. Here, indicate whether each material, system or method listed is relevant to your study. If you are not sure if a list item applies to your research, read the appropriate section before selecting a response.

Materials & experimental systems

n/a	Involved in the study	
☐	☒	Antibodies
☐	☒	Eukaryotic cell lines
☒	☐	Palaeontology and archaeology
☐	☒	Animals and other organisms
☒	☐	Human research participants
☒	☐	Clinical data
☒	☐	Dual use research of concern

Methods

n/a	Involved in the study	
☒	☐	ChIP-seq
☐	☒	Flow cytometry
☒	☐	MRI-based neuroimaging

Antibodies

Antibodies used

The following antibodies were used for flow cytometry:
BD Biosciences: CD20 (clone L27; FITC; Cat# 347673), CD3 (clone SP34-2; PerCP Cy5.5; Cat#552852), IgM (clone G20-127; PE Cy5;551079), CD16 (clone 3G8; PE Cy7; Cat# 557744)
Southern Biotech: IgD (polyclonal; PE: Cat#2030-09)
Invitrogen: CD8 (clone 3B5; PE Texas Red; Cat# MHCD0817)
Biolegend: CD14 (clone M5E2; BV570; Cat# 301832), CD27 (clone O323; APC Cy7; Cat# 302816)

For ELISA and BAMA:
Rockland: HRP-conjugated antibody, polyclonal goat anti-monkey IgG (Cat# 617-103-130)
ThermoFisher: biotinylated Human anti-CD4 (Cat# 13-0048-82), HRP-conjugated Streptavidin (Cat# N100)
Southern Biotech: PE-conjugated mouse anti-monkey IgG (Cat# 4700-09)

Infected cell binding:
Southern Biotech: (FITC)-conjugated goat anti-rhesus IgG(H+L) polyclonal antiserum (Cat# 6200-02)
Beckman Coulter, Inc.: RD1-conjugated anti-p24 MAb KC57 (Cat# 6604667)

Validation

All antibodies were used per manufacturer's instructions and confirmed for reactivity for rhesus monkey samples.

March 2021

Eukaryotic cell lines

Policy information about cell lines

Cell line source(s)	NIH AIDS Reagent Program
Authentication	Cell lines were not authenticated.
Mycoplasma contamination	All cell lines were negative for mycoplasma.
Commonly misidentified lines (See ICLAC register)	N/A

Animals and other organisms

Policy information about studies involving animals; ARRIVE guidelines recommended for reporting animal research

Laboratory animals	Type D retrovirus-, SIV- and STLV-1 free Indian rhesus macaques (Macaca mulatta) were maintained in the colony of California National Primate Research Center (CNPRC, Davis, CA). Adult female rhesus macaques were utilized in this study and ranged from 4 to 10 years of age.
Wild animals	This study did not involve wild animals.
Field-collected samples	This study did not involve samples collected in the field.
Ethics oversight	All protocols were reviewed and approved by the University of California at Davis Institutional Animal Care and Use Committee (IACUC) prior to the initiation of the study.

Note that full information on the approval of the study protocol must also be provided in the manuscript.

Flow Cytometry

Plots

Confirm that:

☒ The axis labels state the marker and fluorochrome used (e.g. CD4-FITC).

☒ The axis scales are clearly visible. Include numbers along axes only for bottom left plot of group (a 'group' is an analysis of identical markers).

☒ All plots are contour plots with outliers or pseudocolor plots.

☒ A numerical value for number of cells or percentage (with statistics) is provided.

Methodology

Sample preparation	Cryopreserved PBMCs were thawed in 37degC H2O bath for approx. 2 minutes. Cells were washed with R10 (RPMI + 10% FBS) + benzoase, and resuspended in R10 for cell counting. 3x10^6 PBMCs were stained according to the manufacturer's guidelines and as described in the Methods section.
Instrument	BD LSRII
Software	FACS DIVA and FlowJo v10
Cell population abundance	At least 1 million events were acquired to account for rare populations.
Gating strategy	Gating strategy is available in the Supplemental Material.

☒ Tick this box to confirm that a figure exemplifying the gating strategy is provided in the Supplementary Information.
